# Elevated Mutation Burdens in Canadian Oat and Wheat Cultivars Released over the Past Century

**DOI:** 10.3390/cells14110844

**Published:** 2025-06-04

**Authors:** Yong-Bi Fu, Carolee Horbach

**Affiliations:** Plant Gene Resources of Canada, Saskatoon Research and Development Centre, Agriculture and Agri-Food Canada, 107 Science Place, Saskatoon, SK S7N 0X2, Canada; carolee.horbach@agr.gc.ca

**Keywords:** deleterious mutation, mutation burden, genetic risk, genetic vulnerability, plant breeding, oat, wheat, breeding gene pool

## Abstract

Modern high-yielding crop cultivars are known to have narrow genetic bases, making them vulnerable to biotic and abiotic stresses. However, little is known about the extent of deleterious genetic variants (or mutation burden) present in these cultivars. An attempt was made using RNA-Seq to screen genome-wide deleterious genetic variants in 141 oat and 142 wheat cultivars released through Canadian breeding programs over the past century. The screening identified 5726 and 3022 deleterious genetic variants across all 21 chromosomes of both the oat and wheat genomes, respectively. These deleterious variants were largely harbored in a few cultivars and were involved with diverse biological processes, cellular components, and molecular functions. More highly deleterious variants were predicted in oat, than in wheat, cultivars, and different gene expression profiles at the early seedling stage were observed between oat and wheat cultivars, illustrating different genetic impacts of the oat and wheat breeding programs. Estimating mutation burdens for each cultivar revealed large variations among both the oat and wheat cultivars. These mutation burdens were found to increase from early to recent oat and wheat cultivars and were associated with higher cultivar yields. Genetic analyses also revealed genetic shifts and expansions from early to recent oat and wheat cultivars. These findings provide the first empirical evidence of elevated mutation burdens in Canadian oat and wheat cultivars and are useful for advancing plant breeding programs to minimize genetic risk.

## 1. Introduction

Deleterious mutation burden measures the extent of deleterious mutations present in an individual. Deleterious mutations are changes in DNA that disrupt normal gene functions and negatively impact an individual’s fitness or ability to survive and reproduce. DNA changes can occur naturally during DNA replication or result from environmental influences such as radiation. Based on the theory of genetic load [1,2], the mutation burden measure should be more informative than other commonly used genetic markers, such as single nucleotide polymorphisms (SNPs), for assessing genetic vulnerability and adaptability to biotic and abiotic stresses at an individual or population level. In fact, this genetic concept has been actively explored in recent human medical research (e.g., [3,4]), thanks to advances in genomics. For example, the number of somatic mutations derived from next-generation sequencing techniques (or tumor mutation burden) has been widely explored as an alternative or complementary biomarker for response to immune checkpoint inhibitors in cancer research (e.g., [5]). However, its application is largely lacking in plant research, particularly in plant breeding and germplasm conservation, and its potential benefits have not received much attention. Deleterious mutations are common in plant genomes. A cultivar or a germplasm accession carrying more deleterious mutations is genetically expected to have higher vulnerability to environmental changes with lower reproduction success and survival [6,7,8], which can limit plant breeding efficacy. Thus, measuring deleterious mutation burden in plant germplasm can provide a better assessment of its genetic vulnerability and is useful for developing breeding strategies for reducing mutation burden in released cultivars.

The last decade has seen increased efforts to screen and report genome-wide deleterious genetic variants present in several plant genomes [9,10,11,12,13,14,15,16,17,18,19,20], largely thanks to mutation screening and analysis in the human genome (e.g., [21,22]) and bioinformatics tools developed for predicting deleterious amino acids from polymorphism (e.g., [23]). Technically, the identification of deleterious variants across a sequenced genome was mainly based on the gene function prediction of a non-synonymous site change alone and/or in combination with the intensity of purifying selection inferred from phylogenetic restraints on the site. These predicted deleterious mutations are more likely to be harmful [7,19,24], although empirical evaluations of their fitness effects are largely lacking [25]. Overall, these efforts have demonstrated the feasibility and value of screening deleterious genetic variants across plant genomes and measuring mutation burden in domesticated populations [6,15,19,20].

Canadian plant breeding programs have played an important role in Canadian agriculture by developing high-yielding crop cultivars with enhanced quality and improved resilience to environmental stresses [26]. For example, Canadian wheat breeding began in 1886 and has released hundreds of cultivars to date [27]. The wheat cultivars released in the last 20 years have up to 6.44 tonnes/hectare grain yields, 150% higher than those (4 tonnes/hectare) developed before the 1950s [28]. Breeding targets have changed from adaptation and quality before 1940, to resistance to biotic and abiotic stresses, such as rust, from 1940 to 1990, to end-use quality, such as increased grain protein, after 1990. The accompanying breeding methods range from introduction, mass selection, hybridization, backcrossing, and marker-based selection, to genomics-assisted breeding. These breeding practices are commonly applied with respect to modern plant breeding and are expected to narrow down the genetic bases of the crop breeding gene pools [29,30]. Little is known, however, about the extent of deleterious mutations harbored in the cultivars released from Canadian plant breeding programs.

We conducted a series of genetic diversity analyses from 1999 to 2009 on existing Canadian gene pools of flax, oat, wheat, soybean, potato, and canola, using different molecular markers (see [31] for a summary). These analyses found that the assayed crop gene pools had variable patterns and degrees of genetic diversity decline over the 100 years of Canadian breeding efforts. Significant allelic loss and genetic shift were also found in the oat and wheat gene pools. This study, however, was configured using the advances in genomics to address a related research question on crop diversity decline: are deleterious mutation burdens in Canadian registered cultivars over the past century associated with their registration years and yields? The specific objectives of the study were (1) to screen and characterize genome-wide deleterious genetic variants in 141 oat (*Avena sativa* L.) and 142 wheat (*Triticum aestivum* L.) cultivars released over the past century from Canadian oat and wheat breeding programs using RNA-Seq [32] and (2) to estimate cultivar-wise mutation burdens to determine their changes over different breeding periods and their associations with cultivar yields. It was our hope that this study would allow for a better understanding of mutation dynamics in existing Canadian breeding gene pools [8] and demonstrate the value of deleterious mutation screening in minimizing genetic risk for plant breeding.

## 2. Materials and Methods

### 2.1. Assayed Oat and Wheat Cultivars

We studied 141 oat cultivars developed and/or registered in Canada between the years 1886 and 2019 (Table S1). Oat seed samples were acquired in December 2019 from Ms. Debbie Nordstrom at Plant Gene Resources of Canada (PGRC), Agriculture and Agri-Food Canada (AAFC), Saskatoon, SK, Canada; Dr. Aaron Beattie at the Crop Development Centre (CDC), University of Saskatchewan, Saskatoon, SK, Canada; and Dr. Jennifer Mitchell Fetch at the AAFC Brandon Research and Development Centre, Brandon, MB, Canada. We also assayed 142 wheat cultivars that were developed and/or registered in Canada between 1845 and 2021 (Table S2). Wheat seed samples were acquired in October 2023 from Ms. Debbie Nordstrom at PGRC; from AAFC plant breeders Dr. Richard Cuthbert at the Swift Current Research and Development Centre, Dr. Harpinder Singh Randhawa at the Lethbridge Research and Development Centre, and Dr. Santosh Kumar at the Brandon Research and Development Centre; and the Canadian wheat breeders Drs. Adam Carter and Pierre Hucl at CDC. Note that the seed samples with CN numbering in Tables S1 and S2 were acquired for this public good research from the PGRC oat and wheat collections, respectively, following the Standard Material Transfer Agreement of the International Treaty on Plant Genetic Resources for Food and Agriculture. These oat and wheat cultivars were selected to represent the cultivars registered over different breeding periods. The oat and wheat breeding gene pools were chosen to represent the long-term Canadian breeding programs.

### 2.2. Cultivar Yield Data Collection

We did not perform any phenotyping on the assayed oat and wheat cultivars, but acquired cultivar yield data from the literature. For oat yields, we collected various sources of oat yield reports (Tables S1 and S1a), but mainly focused on the 5-year average yields of the assayed cultivars reported from SCIC Oats Provincial Average Yields by Variety (2019–2023) data (https://www.producer.com/digital-edition/yield-saskatchewan/2024-03-20; accessed on 25 February 2025). For cultivars not included in the SCIC report, other published sources, which shared some cultivars with SCIC, were used to calculate adjusted cultivar yields. The yield adjustment of a cultivar absent in one report was based on its yield in another report, along with the yields of another cultivar present in both reports. For example, SCIC reported yield for CDC Endure and not for CDC Skye, while the *Seed Manitoba Variety Selection* & *Growers Source Guide* (2022; or SMV) had yield reports on both CDC Endure and CDC Skye. The adjusted yield for CDC Skye is (Yield_CDCSkye-SMV_/Yield_CDCEndure-SMV_) × Yield_CDCEndure-SCIC_, assuming both cultivars have the same yield performance in the fields from both evaluations. The yield reports and cultivars used for yield adjustment are listed in Table S1a. The effort generated yield data for 92 assayed cultivars (Table S1) and all yield data was converted to bushels per acre (bu/ac) based on a standard weight of 32 pounds per bushel for oats (https://www.smallfarmcanada.ca/resources/standard-weights-per-bushel-for-agricultural-commodities; accessed on 25 February 2025).

For wheat yields, a similar effort was made to collect yield data for 105 wheat cultivars from various Canadian wheat yield reports (Tables S2 and S2a). The primary report was yield data published by Iqbal et al. [28]. The secondary reports included SCIC Wheat-Hard Red Spring Provincial Average Yields by Variety (2018–2022) (https://www.producer.com/digital-edition/yield-saskatchewan/2023-04-01; accessed on 25 February 2025) and the *Seed Manitoba Variety Selection & Growers Source Guide* (2022) (https://www.seedmb.ca/wp-content/uploads/2021/11/SMB_2022.pdf, accessed on 25 February 2025). These two reports, along with several other publications, were used to adjust yields with respect to those from Iqbal et al. [28]. The same adjustment as for oat cultivars was used for wheat cultivars absent in Iqbal et al.’s yield data. However, the secondary wheat yield reports shared several wheat cultivars, and thus, the adjusted yields for some of the cultivars absent in Iqbal et al.’s data were generated based on the average of the yields of CDC Go, Carberry, and CDC Stanley from each report adjusted to Iqbal et al. Selecting these three cultivars for adjustment was arbitrary but doing so helped minimize yield variation within and among yield reports. The yield reports and cultivars used for yield adjustment are listed in Table S2a. All yield data was converted to bushels per acre (bu/ac) based on a standard weight of 60 pounds per bushel for wheat (https://www.smallfarmcanada.ca/resources/standard-weights-per-bushel-for-agricultural-commodities; accessed on 25 February 2025).

### 2.3. RNA-Seq Analysis

Up to 10 seeds per cultivar were planted in 48-well seeding trays with PRO-MIX BX soil (Premier Tech, Rivière-du-Loup, QC, Canada) in the greenhouse at the Saskatoon Research and Development Centre, with conditions of 22 °C for 16 h day and 16 °C for 8 h night. When the seedlings reached the 3-leaf stage, approximately 2 cm of tissue was collected separately from each plant from the region just above the root collar, containing the apical meristem. Tissue was placed in 2 mL tubes, snap-frozen in liquid nitrogen, and stored at −80 °C until RNA extraction.

RNA was extracted from the leaf tissue of a single plant per cultivar using a Qiagen RNeasy Plant Mini Kit (Qiagen Inc., Toronto, ON, Canada) with buffer RLT, following the provided protocol. The final wash with buffer RPE was repeated to reduce guanidine thiocyanate carryover. All extracted RNA samples were treated with an Invitrogen DNA-free^TM^ DNA Removal Kit (Thermo Fisher Scientific, Waltham, MA, USA). RNA was quantified using a Thermo Scientific Nanodrop 8000 (Thermo Fisher Scientific, Waltham, MA, USA) and its quality was assessed with randomly selected samples using a Bioanalyzer RNA 6000 Nano Chip (Agilent, Santa Clara, CA, USA). Extracted RNA samples were stored at −80 °C. Sequencing libraries were generated using an NEBNext Ultra II Directional RNA Library Prep Kit for Illumina with an NEBNext Poly(A) mRNA Magnetic Isolation Module (New England Biolabs, Whitby, ON, Canada), following the instructions provided with the kit, which included modifications for insert sizes of approximately 300 bp. Libraries were pooled and sequenced at the Centre d’expertise et de services Génome Québec on one lane of an Illumina NovaSeq with 100 bp paired-end reads. The acquired raw sequences were deposited in 2024 to the National Center for Biotechnology Information Sequence Read Archive database under BioProject ID PRJNA1132186 for the oat cultivars and PRJNA1135233 for the wheat cultivars. Note that the RNA-Seq analyses were conducted on oat samples in early 2021 and on wheat samples in the summer of 2024.

### 2.4. SNP Calling

A pair of demultiplexed forward and reverse FASTQ files were generated for each sample. FastQC [33] was used to assess the overall sequencing quality of each sample. FASTQ files were trimmed with Trimmomatic v0.32 [34] to remove any adapter sequences, low-quality sequences (below a Phred score of 24), and any sequences shorter than 80 bases. The following trim settings were used: ILLUMINACLIP: TruSeq3-PE-2.fa; SLIDINGWINDOW:10:24; and MINLEN:80. FastQC was re-run after trimming to verify the removal of the Illumina adapter sequences. The reference genome sequences for oat and wheat were Asativa_sang_pseudomolecules_0.zip [35] and Triticum_aestivum.IWGSC.dna_rm.toplevel.fa.gz [36], respectively. These reference genomes were split using a custom Perl script [18], as the number of bases in each chromosome FASTA entry must be below the 2^39^−1 base (≈536 Mb) limit of Samtools v1.6 [37] BAM indexing. The sample FASTQ files were aligned against the reference genome sequence using the Burrows–Wheeler Aligner v0.7.17 [38] BWA-MEM algorithm. The resulting BAM files were filtered to remove PCR duplicates using the MarkDuplicates tool from the Genome Analysis Toolkit v4.2.6.1 [39]. Samtools sort was applied to produce sorted BAM files. SNP calls were performed using Bcftools v1.9 with the following command: bcftools mpileup -Ou -f -b | bcftools call -vmO z -V indels -o. To speed up the computation, SNPs were called separately for each chromosome and the resulting SNP VCF files were concatenated using Bcftools concat function. The concatenated VCF file with split chromosomes was combined back into full chromosomes using a custom Perl script [18]. SNP quality filtering was performed using Vcftools v0.1.15 [40] with the following command: vcftools –vcf input.coord.vcf –out output.coord.vcf –recode-INFO-all –max-alleles 2 –min-alleles 2 –minDP 10 –minQ 20 –max-missing 1 –recode. This SNP calling procedure was performed for both oat and wheat samples.

### 2.5. Identification of Deleterious SNPs

The generated SNP VCF files were used to perform SNP annotations with the stand-alone Ensembl Variant Effect Predictor (VEP) [41,42]. The sorting intolerant from tolerant (SIFT) algorithm [43] was applied to predict the deleterious effect of every identified genetic variant for its gene function with a SIFT score. The SIFT score can distinguish between functionally neutral and deleterious amino acid changes. An amino acid substitution with a SIFT score of 0.05 or smaller is predicted to be deleterious. The VEP analysis of the wheat VCF file generated SIFT scores directly for all variants, but for the oat SNPs, a separate SIFT analysis was performed based on a previously generated oat SIFT database [18]. These efforts generated a SIFT score for each SNP for the oat and wheat samples. To increase the accuracy of identifying deleterious SNPs (dSNPs), this study applied both SIFT score and GERP++ Rejected Substitution (RS) score [44] to evaluate a SNP. The RS scores for the extremely conserved chromosomal regions of oat and wheat genomes were previously generated using GERP++ based on reference genomes of 12 plant species to measure the phylogenetic constraint from the substitution of a locus based on 12 plant species [18]. The resulting RS score provides a quantification of the conservation of each nucleotide in a multi-species alignment. A positive score (RS > 0) at a substitution site means fewer substitutions than expected. Thus, a substitution occurring in a conserved site with RS > 0 is predicted to be deleterious; the larger the RS score, the more deleterious the substitution. Specifically for this study, SIFT (<0.05) and GERP++ RS (>0) annotations were combined to identify dSNPs in constrained portions of the genome. The identified dSNPs were further classified to be weakly, mildly, and highly deleterious based on GERP++ RS scores of <1, 1–3, and >3, respectively. Note that a dSNP (or mutation) in this study is defined as a SNP (or mutation) predicted to be deleterious to its gene function, not necessarily to overall plant fitness. Based on the previous studies [7,19,24], the genetic variants identified with the approaches used in this study are more likely to be harmful.

With the identification of dSNPs, the genotyping of all the assayed samples at the dSNP loci was performed to estimate mutation burdens. The original deleterious SNP genotype VCF files generated from this study were also included in the Supplemental Materials for future uses. Fixed dSNPs were identified based on the allelic frequency data for all assayed cultivars of each crop. The dSNPs and total detected SNPs were counted for each chromosome to compare their distributions over each genome, and their allelic frequencies in all the assayed cultivars were also analyzed.

### 2.6. Gene Ontology (GO) and Expression Analysis

GO analysis of the predicted dSNPs started with the extraction of genes associated with the identified dSNPs from species gene annotation files. Then, the nucleotide FASTA data for these associated genes was extracted from the species assembly cds FASTA files. These gene nucleotide FASTA files were uploaded to the Galaxy server (https://usegalaxy.org/, accessed 1 June 2025) for gene enrichment analysis using eggNOG-mapper v5.0.2 [45] to generate GO annotations based on precomputed orthologous groups and phylogenies from the eggNOG database. The orthology-based eggNOG-mapper is expected to be more accurate in GO annotations than other GO analytic tools. The resulting GO term sets were further analyzed and visualized using g:Profiler version *e112_eg59_p19_25aa4782* [46] with multiquery plots and REVIGO v1.8.1 [47] with treemaps and tag clouds to aid the interpretation of gene enrichments and functions.

Gene expression analysis of genes associated with the identified dSNPs was conducted by counting sequence reads for each associated gene. This was carried out for each sample using the StringTie program [48] from oat or wheat RNA-Seq data. The sample-wise gene expression data at the early seedling stage was further correlated with the cultivar registration years of each crop by linear regression analysis with the R lm function [49].

### 2.7. Mutation Burden Estimation and Its Association with Cultivar Features

Sample dSNP genotype data were generated or extracted based on the identified dSNPs from each crop SNP VCF file. The mutation burden per deleterious locus was estimated for each sample based on its number of deleterious alleles [15]. Three models were considered in this study: homozygous mutation burden, heterozygous mutation burden, and total mutation burden. The homozygous mutation burden per deleterious locus is defined as the number of derived deleterious alleles in the homozygous state, divided by a product of 2 × total dSNP count. The heterozygous mutation burden per deleterious locus is calculated as the number of derived deleterious alleles existing in the heterozygous state, divided by a product of 2 × total dSNP count. The total mutation burden per deleterious locus is equal to the number of derived deleterious alleles existing in a sample (2 × homozygous mutation burden + heterozygous mutation burden), divided by a product of 2 × total dSNP count. These burdens per deleterious locus were estimated for each cultivar of both crops.

A linear regression analysis was conducted using the R lm function of the estimates of a mutation burden per deleterious locus (total, heterozygous, or homozygous) over the cultivar registration years and the available cultivar yields. The results were plotted using the R plot function [49]. These analyses were repeated for the combination of the three mutation burdens per deleterious locus with cultivar yield and registration year of the two crops.

### 2.8. Nucleotide Diversity and Genetic Association Analysis

To understand the genetic changes in the registered cultivars over different breeding periods, the assayed cultivars were first grouped (Tables S1 and S2) based on the periods of their registrations following the previous oat [50] and wheat [51] genetic diversity studies. The nucleotide diversity per site was estimated across a chromosome for a given period of registration using Vcftools, with the option of site-pi based on the SNP VCF file of oat or wheat cultivars. The estimates for all 21 chromosomes were averaged and their variations were quantified for the breeding periods. Such an estimation was performed for each of the 10 oat breeding periods and 9 wheat breeding periods.

The genetic associations of the assayed oat and wheat cultivars were analyzed using R package SNPRelate [52] with its function of snpgdsPCA based on the SNP VCF file of oat or wheat cultivars. The function was executed to perform the principal component analysis (PCA) of SNP data and generate eigenvectors and their variance proportions. PCA plots were made using R package ggplot2 [53] with different sample labels for various breeding periods. This analysis was performed separately for oat and wheat cultivars.

## 3. Results

### 3.1. SNP Identification and Annotation

RNA-Seq sequencing produced a total of 2922 million sequence reads for oat samples with an average of 28 million mapped sequence reads per sample (Table S3) and 3560 million sequence reads for wheat samples with an average of 46.5 million mapped sequence reads per sample (Table S4). SNP calling from RNA-Seq data identified 4,027,667 and 4,504,626 SNPs for oat and wheat samples, respectively. After SNP quality filtering and missing value removal, there were 253,264 oat and 270,622 wheat SNPs remaining for further analysis (Table 1). These identified SNPs were widely distributed across 21 oat and wheat chromosomes (Figure S1A,C). Specifically, the SNP count per chromosome ranged from 5565 to 20,265 with an average of 12,060.2 for the oat samples and from 6114 to 19,102 with an average of 12,695.3 for the wheat samples. These SNPs also displayed expected L-shape distributions of minor allele frequency for oat and wheat samples (Figure S2), with one exception that there were higher than expected invariant heterozygotes with a minor allele frequency of close to 0.5 in the wheat samples (Figure S2A2).

VEP-based annotation analyses of the identified SNPs allowed for the classification of SNPs into 15 and 17 different classes with the most severe consequences for oat and wheat samples (Table 1), respectively. For the oat samples, the classes with the most SNPs were downstream_gene_variant (131,064), followed by synonymous_variant (125,064), upstream_gene_variant (83,198), missense_variant (74,655), 3_prime_UTR_variant (52,173), and 5_prime_UTR_variant (14,382). Similarly for the wheat samples, the classes with the most SNPs were synonymous variant (144,071), followed by downstream_gene_variant (114,488), missense_variant (94,003), upstream_gene_variant (71,843), 3_prime_UTR_variant (57,649), and 5_prime_UTR_variant (29,115). The proportions of missense variants and loss-of-function variants over all the identified SNPs were 0.295 and 0.0076, respectively, for the oat samples. Similarly, the proportions of missense variants and loss-of-function variants over all the identified SNPs were 0.347 and 0.0097, respectively, for the wheat samples.

### 3.2. Deleterious Mutation

dSNPs were identified first with SIFT scores and later in combination with SIFT and RS scores. Analyzing SIFT scores for all non-synonymous SNPs revealed 16,208 and 17,519 SNPs being deleterious in the oat and wheat samples, respectively. After excluding the SNPs with deleterious_low_confidence, the dSNPs were reduced to 12,182 and 12,855 in the oat and wheat samples, respectively (Table 1). The proportion of the total SNPs being deleterious based on SIFT scores was found to be 0.0481 and 0.0475 for the oat and wheat samples, respectively (Table 1). Combining SIFT scores with RS scores identified 5726 and 3022 SNPs as being deleterious for the oat and wheat samples, respectively (Table 1). The proportions of the detected dSNPs for the oat and wheat samples were 0.0226 and 0.0112, respectively. Based on the frequencies of dSNPs in all assayed samples of a crop, there were 3 and 16 dSNPs being fixed for the oat and wheat samples, respectively.

The dSNPs identified from SIFT and RS scores were widely distributed across 21 oat and wheat chromosomes (Figures S1B and S1D, respectively). Specifically, the SNP count per chromosome ranged from 137 to 428 with an average of 272.7 for the oat samples, and from 76 to 242 with an average of 143.9 for the wheat samples. These dSNPs also displayed expected L-shape distributions of minor allele frequency for the oat and wheat samples (Figure S2B). Specifically, there were 3490 (61%) oat dSNPs with minor allelic frequencies of 0.05 or less (or present in seven or fewer samples) and 1300 (22.7%) with 0.01 or less (or present in one sample only). Similarly, there were 1663 (50%) wheat dSNPs with minor allelic frequencies of 0.05 or less and 745 (24.7%) with 0.01 or less. Thus, most of the identified dSNPs were harbored in only a few oat and wheat samples.

The identified dSNPs could be classified to be weakly, mildly, and highly deleterious based on GERP++ RS scores <1, 1–3, and >3 (Table 1; Figure S3), respectively. Specifically, there were 2348 (41%) weakly, 2295 (40.1%) mildly, and 1083 (18.9%) highly deleterious SNPs in the oat cultivars, while there were 2834 (93.8%) weakly, 161 (5.3%) mildly, and 27 (0.9%) highly deleterious SNPs in the wheat cultivars. Thus, there were more predicted, highly deleterious SNPs identified in the oat, than in the wheat, cultivars.

### 3.3. Ontology and Expression of the Associated Genes

**Figure 1:**
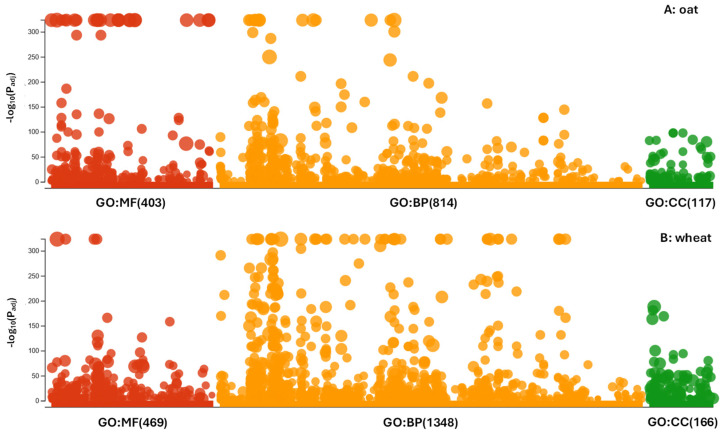
g:GOSt multiquery Manhattan plots showing the amounts of molecular functions (MF), biological processes (BP), and cellular components (CC) for 585 significant oat GO terms (**A**) and 262 significant wheat GO terms (**B**).

**Figure 2:**
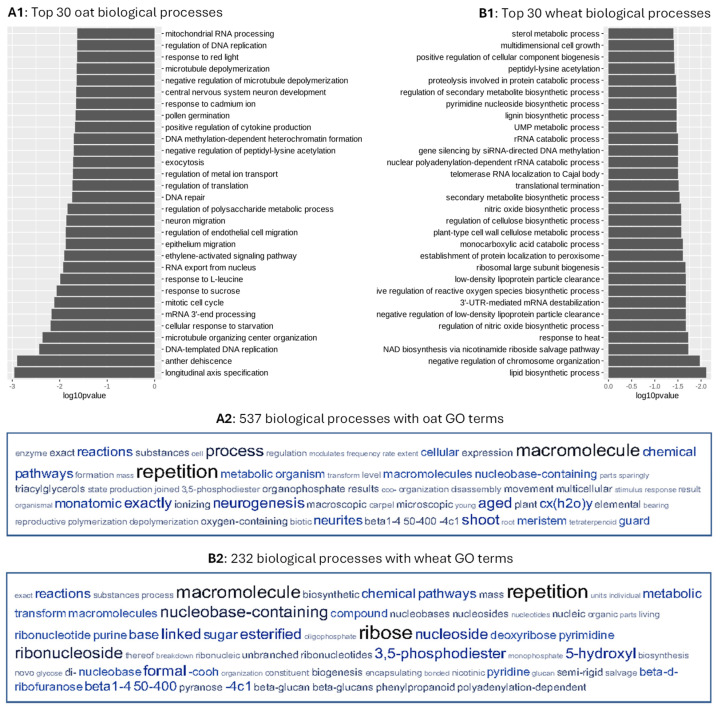
Top 30 oat (**A1**) and wheat (**B1**) biological processes with their significant GO terms and tag word clouds of 537 biological processes with 585 significant oat GO terms (**A2**) and 232 biological processes with 262 significant wheat GO teams (**B2**), as revealed from the REVIGO analysis. The significant GO terms were identified from 7157 oat and 3533 wheat genes associated with the identified deleterious SNPs, respectively. The top 30 biological processes were unique to oat or wheat and two obvious tag words (macromolecule and repetition) were shared between the two word clouds.

The identified dSNPs were found to be associated with 7157 and 3533 genes across all chromosomes for the assayed oat and wheat cultivars, respectively. The ontology analyses of the associated genes identified 585 significant oat GO terms and 262 significant wheat GO terms (*p* < 0.05). Analysis of these GO terms using g:Profiler revealed 403 molecular functions, 814 biological processes, and 117 cellular components for the associated oat genes, and 469 molecular functions, 1348 biological processes, and 166 cellular components for the associated wheat genes (Figure 1). By reducing the redundant GO terms, REVIGO still revealed 537 biological processes in oats (Figure S4) and 232 biological processes in wheat (Figure S5). The oat biological processes were mainly involved with cellular response to amino acid stimulus, negative regulation of axonogenesis, RNA secondary structure unwinding, unidimensional cell growth, DNA duplex unwinding, mitochondria translation, intra-Golgi vesicle-mediated transport, lipid phosphorylation, and regulation of exocyst localization. Similarly, the wheat biological processes were mainly associated with nicotinamide nucleotide biosynthetic process, regulation of beta-glucan biosynthetic process, nuclear pore complex assembly, telomere tethering at nuclear periphery, peptidyl-amino acid modification, response to cold, cell development, and polyketide metabolic process. The top 30 oat and wheat biological processes were shown in Figure 2(A1,B1), respectively. Interestingly, these top 30 processes were unique to oat or wheat. The first two processes in oat were longitudinal axis specification and anther dehiscence. In wheat, lipid biosynthetic process and negative regulation of chromosome organization were the first two processes. The revealed oat and wheat biological processes shared two tag words: macromolecule and repetition (Figure 2(A2,B2)). However, further analyses of the 117 oat and 166 wheat cellular components generated from g:Profiler revealed 63 shared cellular components such as cell wall, cell periphery, cytoplasm, and transporter complex. As shown in Figure S6, out of the top 40 cellular components in oat and wheat, 19 are shared between them. Similarly, some shared molecular functions were also found, as illustrated with four out of the top 40 molecular functions (Figure S7).

Analyzing the amounts of sequence reads for the associated genes expressed in each sample revealed large variations in transcripts per million per gene among all expressed genes in both oat (Table S5) and wheat (Table S6) samples. Averaging the transcripts per million per gene across all the genes also showed large variations among oat samples ranging from 4.81 (Gold Rain) to 6.44 (Stainless) and among wheat samples from 8.11 (AAC Hodge) to 11.84 (AC Barrie). The linear regression analyses of the gene expression data associated with dSNPs over the cultivar registration years revealed significantly (*p* < 0.001) increased expressions of the associated genes at the early seedling stage from early to recent oat cultivars (Figure S8A), and a marginally significant (*p* < 0.06) decrease in expressions of the associated genes from early to recent wheat cultivars (Figure S8B).

### 3.4. Mutation Burden

**Figure 3:**
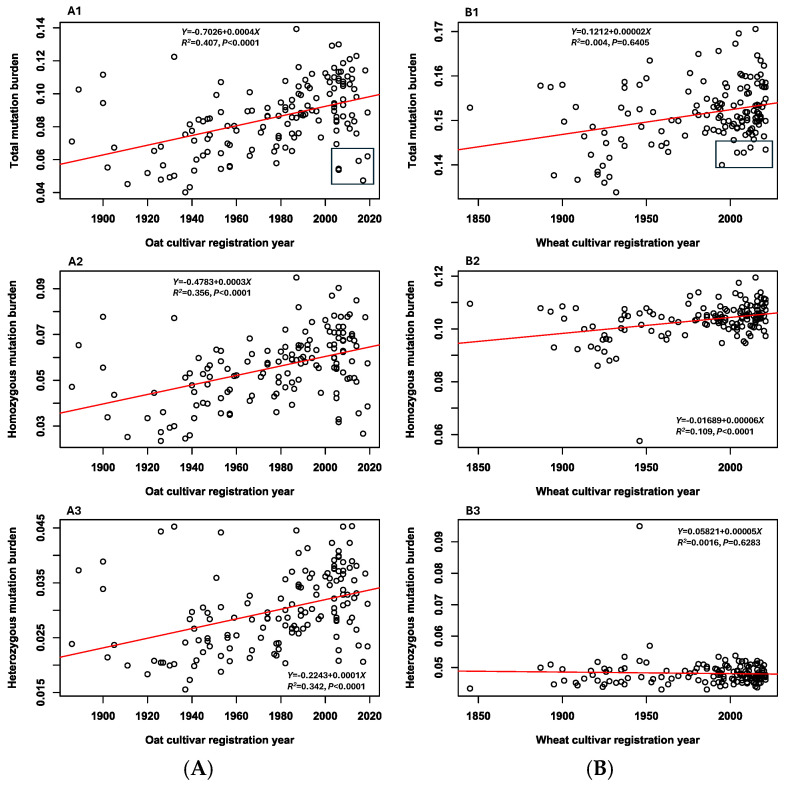
The associations of three mutation burden estimates per deleterious locus with the registration years of the 141 oat (**A**) and 142 wheat (**B**) cultivars. Five oat cultivars in the box of (**A1**) (released after 2005) with lower total mutation burden estimates are CDC Arborg (0.047), Domingo (0.054), Bia (0.055), CDC Norseman (0.059), and CDC Endure (0.062). Five wheat cultivars in the box of (**B1**) (released after 1994) with lower total mutation burden estimates are Prodigy (0.140), Stettler (0.143), Infinity (0.143), AAC Rimbey (0.143) and CDC VR Morris (0.144).

Estimates of three mutation burdens (total, heterozygous, and homozygous) per deleterious locus were made for each sample and presented in Tables S5 and S6 for the oat and wheat cultivars, respectively. Large variations in the three mutation burden estimates were observed in oats (Figure S9A). For example, the estimates of total individual mutation burden varied from 0.040 to 0.139 with a mean of 0.086 for the oat cultivars (Table S5). The oat cultivar with the highest total mutation burden estimate was Robert (0.139), followed by CDC SO-I (0.130), Furlong (0.129), AAC Oaklin (0.123), and Bell (0.122). The cultivar with the lowest total mutation burden estimate was Eagle (0.040), followed by Lanark-1 (0.043), Victory (0.045), CDC Arborg (0.047), and Gold Rain (0.048). Figure 3(A1) showed the five oat cultivars released after 2005 with lower mutation burdens: CDC Arborg (0.047), Domingo (0.054), Bia (0.055), CDC Norseman (0.059) and CDC Endure (0.062). In contrast for the wheat cultivars, only the variation in homozygous mutation burden estimates was relatively large (Figure S9B3), while the heterozygous and total mutation burden estimates showed little variation among the cultivars (Figures S9B1 and S8B2). The total mutation burden estimate per wheat sample ranged from 0.134 to 0.171 with a mean of 0.152 (Table S6). The five wheat cultivars with the highest total mutation burden estimates were AAC Connery (0.171), Somerset (0.170), BW776 (Lillian) (0.167), AC Eatonia (0.166), and Leader (0.165). The five wheat cultivars with the lowest total mutation burden estimates were Reliance (0.134), Broatch’s Whitehead (0.136), Marquis (0.137), Ceres (0.137), and Preston (0.138). The five wheat cultivars released after 1994 with lower total mutation burden estimates were Prodigy (0.140), Stettler (0.143), Infinity (0.143), AAC Rimbey (0.143), and CDC VR Morris (0.144) (Figure 3(B1)).

### 3.5. Associations Between Mutation Burdens and Cultivar Features

**Figure 4:**
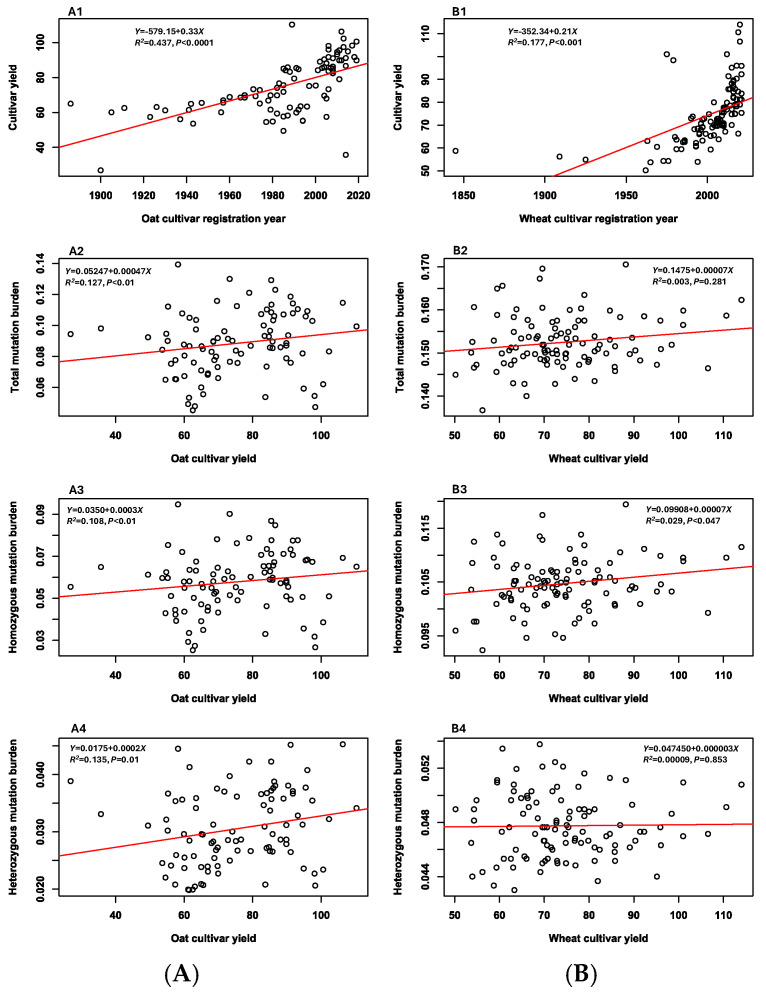
The associations of cultivar yield and three mutation burden estimates with the registration years of the 141 oat (**A**) and 142 wheat (**B**) cultivars.

The linear regression analyses of three mutation burden estimates per sample (total, homozygous, and heterozygous) over the years of oat cultivar registration revealed significant (*p* < 0.0001) increases from early to recent oat cultivars (Figure 3A). However, similar regression analyses of the wheat cultivars showed only a significant (*p* < 0.0001) increase in homozygous mutation burden estimates per sample from early to recent wheat cultivars (Figure 3(B2)), while there were only trends of increasing total and heterozygous mutation burden estimates from early to recent wheat cultivars (Figure 3(B1) and Figure 3(B3), respectively). Further regression analyses of the three mutation burden estimates over the oat cultivar yields (bu/ac) revealed significant (*p* < 0.01) correlations (Figure 4(A2–A4)). The higher the oat cultivar yields, the more mutation burdens accumulated in the oat cultivars. The similar marginally significant (*p* < 0.047) correlation was also found between homozygous mutation burden estimates and wheat cultivar yields (Figure 4(B3)), while there was only the trend of increasing total and heterozygous mutation burden estimates with higher wheat cultivar yields (Figure 4(B2,B4)). Together, these two sets of regression analyses provided empirical evidence that the modern Canadian oat and wheat breeding programs had significantly improved oat and wheat cultivar yields, but also accumulated substantial mutation burdens in the existing breeding gene pools.

### 3.6. Nucleotide Diversity and Genetic Association

**Figure 5:**
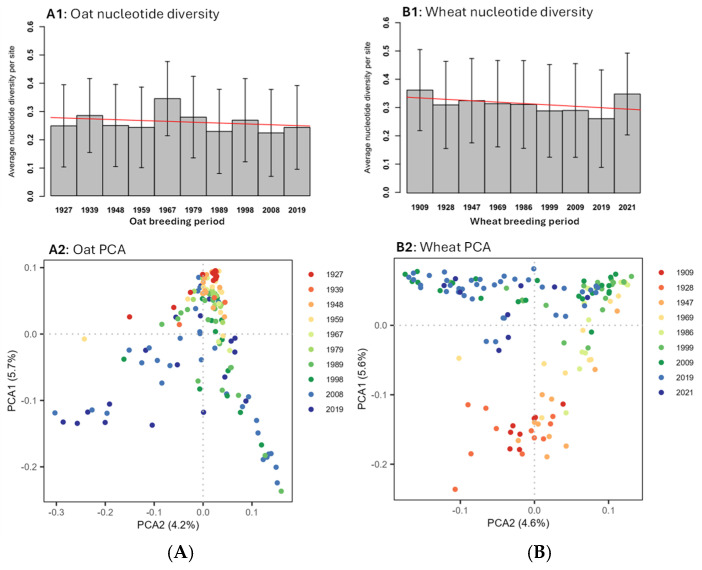
Estimates of average nucleotide diversity per site and the genetic associations of 141 oat cultivars (**A**) over 10 breeding periods and 142 wheat cultivars (**B**) over 9 breeding periods. Both linear regression lines (in red) of nucleotide diversity estimates over the accumulative periods counted from 1 to 10 (for oat) or 9 (for wheat) were not statistically significant at *p* < 0.05, but showed the trend of declining nucleotide diversity per site in both released oat and wheat cultivars from the early to recent periods. The genetic associations were revealed by the principal component analysis of their genome-wide SNPs. Each period was represented by the cultivar(s) with the last year of its period. Genetic shift and expansion were observed among the oat and wheat cultivars from the early breeding periods.

The average estimates of nucleotide diversity per site for different breeding periods of oat and wheat cultivars were plotted in Figure 5. These diversity estimates varied across different breeding periods and had large standard deviations across all 21 chromosomes for each breeding period. Linear regressions of these diversity estimates over the accumulative breeding periods were not statistically significant at *p* < 0.05. However, there was an obvious trend of declining nucleotide diversity per site in both oat and wheat cultivars from early to recent breeding periods (Figure 5(A1,B1)).

The genetic associations of both oat and wheat cultivars as revealed by the principal component analysis of their genome-wide SNPs had two major features (Figure 5(A2,B2)). First, genetic shift was found in both oat and wheat cultivars from early to recent breeding periods. Second, the genetic background in both oat and wheat cultivars was also expanded from early to recent breeding periods. The expansion was much more substantial in the oat, than in the wheat, cultivars, as the earliest period of oat cultivars had an extremely narrow genetic base. Together, these diversity and association findings suggest that the genetic backgrounds of the existing oat and wheat breeding gene pools were expanding with introductions of new germplasm over the years, but their genomic diversities were still narrowing due to the repeated use of some elite parental lines during breeding. The narrowed genomic diversity implies some limit in the genetic improvement of some traits of breeding interest.

## 4. Discussion

This study represents the first genomic screening of deleterious genetic variants in existing Canadian oat and wheat breeding gene pools and revealed several interesting findings. First, a large number of deleterious genetic variants were found to be widely distributed across every chromosome of the oat and wheat genomes. These deleterious variants were largely harbored in only a few cultivars and were involved with diverse biological processes, cellular components, and molecular functions. More highly deleterious variants were predicted in the oat, than in the wheat, cultivars, and different gene expression profiles at the early seedling stage were observed between oat and wheat cultivars, illustrating different genetic impacts of the oat and wheat breeding programs. Second, the estimated mutation burdens varied among the oat and wheat cultivars. These burdens were found to increase from early to recent oat and wheat cultivars and were associated with increased cultivar yields. Third, genetic shifts and expansions were also found from early to recent oat and wheat cultivars. These findings are significant, as they provide the first empirical evidence of elevated mutation burdens in the Canadian oat and wheat breeding gene pools and are useful for advancing plant breeding programs to minimize genetic risk.

The finding of elevated mutation burdens over breeding periods (Figure 3) is largely expected genetically when considering the intensive artificial selection operating over long-term breeding within the narrow gene pool of a selfing crop [6,8,54]. Selfing in breeding can effectively purge deleterious mutations with large effects, but weaker mutations are less likely to be eliminated [55,56]. Repeated uses of a few elite parental lines during breeding, as implied by the narrowed genomic diversity (Figure 5(A1,B1)), could have led to an accumulation of similar deleterious variants within the breeding gene pools. More importantly, however, the finding provides additional empirical support for the long-standing concern of reduced crop genetic diversity on genetic vulnerability and adaptability to changing environments (e.g., [30,31,57,58,59]). The declining trend of nucleotide diversity per site in oat or wheat cultivars (Figure 4) is aligned well with the previous reports of reduced genetic diversity in oat [50] or wheat [29,51] cultivars.

The results of the mutation burden increasing with higher oat and wheat cultivar yields (Figure 4) are interesting, as selecting high-yielding and disease-resistant genes through multiple cycles of selfing was not effective in purging all deleterious variants. Such a low efficiency of purging is not surprising, as the detected variants were largely those of weakly or mildly deleterious effects, as evidenced in Table 1 [54,55,56]. These weakly deleterious variants may not have large impacts on cultivar yield and/or disease resistance, but may negatively influence other traits, especially those not directly targeted in breeding [60]. Thus, these results raise a new challenge for plant breeding and necessitate the development of some strategies to reduce the mutation burden in a breeding program [20,61]. While the oat and wheat gene pools are genetically narrow, it is encouraging to confirm genetic shift and expansions detected in the assayed gene pools (Figure 5; [29,50,51]), as the breeding efforts over the last century have introduced new germplasm and enlarged the genetic backgrounds of these gene pools. Overall, the increase in mutation burden with cultivar registration year and yield demonstrates that, although Canadian oat and wheat breeding programs over the last century significantly improved cultivar yields and expanded genetic backgrounds of the breeding gene pools, they also accumulated more deleterious mutations and reduced genomic nucleotide diversity, making the released cultivars vulnerable to changing environments, particularly under global warming.

This study identified a compatible level of SIFT-based deleterious SNPs for oat and wheat cultivars (12,182 vs. 12,855, respectively), but a much higher number of deleterious SNPs in oat (5726) than wheat (3022) cultivars, when GERP++ RS scores were considered together. It is possible that more extremely conserved regions were identified by GERP++ in the oat genome of size 10.8 Gb than in the wheat genome of size 17 Gb. Comparison with previous research [18] revealed that the proportion of deleterious SNPs detected over all the identified SNPs in oat cultivars (0.0226) was compatible with those (0.0285) inferred from conserved oat germplasm, but, in wheat cultivars, was higher (0.0112) than those inferred from conserved wheat germplasm (0.0034) [18]. This discrepancy may have arisen from the use of different SNP calling procedures with much fewer (or 270,622) SNPs without missing values in the wheat cultivars than the extent of 1,684,050 SNPs identified in the diverse 72 wheat germplasm accessions of the previous study [18]. However, the average individual total mutation burden estimates (0.086 and 0.152) in this study were compatible with those inferred from the conserved oat and wheat germplasm (0.069 and 0.170), respectively [18].

The analyses presented here have some weaknesses worth mentioning. First, the sequencing was based on RNA extracted at the early seedling stage. The analysis considered only the expressed or transcribed deleterious mutations and the findings may be completely specific to a developmental stage. Thus, it would be desirable to evaluate the variability in the identification and characterization of deleterious mutations among other tissues and developmental stages. Also, the analyses assayed only a single plant per cultivar and did not consider the variability of deleterious mutations among plants of a cultivar. However, such mutation variability should still exist within a cultivar of a selfing crop, as demonstrated in barley germplasm [62]. Second, the mutation identification was dependent on the quality of sequencing data, assembled reference genome, bioinformatic tools used for mutation screening, and sample size. Thus, biases should exist in these mutation inferences and comparisons. Third, the deleterious mutations reported here are more likely to be harmful [7,19,24], but are still predictive in nature. It remains unknown if these deleterious mutations were associated with genes conditioning traits of breeding targets such as yield or disease resistance and/or genes influencing plant adaptability and survival. Further research is still needed on the plant fitness consequences of these predicted deleterious mutations on the assayed cultivars. Fourth, our collection of the cultivar yields with adjustments from the related literature carried some assumptions of being equal in growth and survival performances among the assayed cultivars over different sites in different years. Thus, the acquired yields can vary and be biased by the selection of different cultivars for adjustment and/or the use of different adjustment scales. It is difficult to determine the accuracy and bias of these yield adjustments. Clearly, a field trial with a proper experimental design would be more informative for a mutation-yield association analysis.

In spite of these weaknesses, the identified deleterious genetic variants have several interesting features within and between two breeding gene pools, allowing for a better understanding of the long-term genetic impacts of oat and wheat breeding programs. The identified deleterious variants were widely distributed across 21 chromosomes (Figure S1) and largely had low frequencies or occurred only in a few cultivars (Figure S2). Most of the identified deleterious variants were predicted to be weakly and mildly deleterious (Figure S3), but the oat cultivars carried much more highly deleterious variants than the wheat cultivars (Table 1). The deleterious variants were associated with genes conditioning diverse sets of biological processes in both oat (Figure S4) and wheat (Figure S5) breeding gene pools. Interestingly, these revealed biological processes were not always shared between two gene pools (Figure 2), indicating different genetic impacts of similar breeding practices in oats and wheats. However, more than half of the identified oat cellular components were shared with those wheat cellular components (Figure S6). More interestingly, the genes associated with these deleterious variants had higher gene expressions at the early seedling stage in the recent, than in the early, oat cultivars (Figure S8A). In contrast, a decreasing pattern of gene expression for genes associated with wheat deleterious variants was observed from the early to recent wheat cultivars (Figure S8B). It would be interesting to determine how general these features are with respect to a breeding gene pool [19]. Also, it is important to study the impacts of these deleterious variants on plant cell biology, particularly cellular responses to various stresses, as understanding plant cellular mechanisms involved in these responses can provide guidance for developing strategies to mitigate genetic vulnerability and improve plant resilience.

The findings presented here have some practical implications for plant breeding programs. First, the finding of the mutation burden increases over the past century clearly demonstrates the elevated genetic cost of modern plant breeding. The burden increase will lead to cultivars with a higher genetic risk of being vulnerable and reducing adaptability to biotic and abiotic stresses in changing environments [6,8,63]. As mentioned above, it is important to develop breeding strategies for mitigating genetic risks in a breeding gene pool, particularly for a long-term breeding program [20,60,61]. One simple strategy is to evaluate deleterious mutation burdens of elite lines in an active breeding gene pool to provide additional guidance on the parental selection of elite germplasm with the lowest possible mutation burdens. For example, the five oat and wheat cultivars released in recent decades and identified with lower mutation burdens from this study (Figure 3(A1,B1)) could be considered as candidate lines to explore, along with other selection elements in Canadian oat and wheat breeding. Second, genomic mapping of deleterious mutations is technically possible and practically feasible for a plant breeding program, as demonstrated in this study and others (e.g., [20]). In this study, the RNA-Seq analysis of 141 oat or 142 wheat cultivars had an experimental cost in 2024 of CAD 18,000 (including RNA extraction kit, library preparation kit, and sequencing) and the whole mutation screening can be completed within three months from seed planting, sequencing, to mutation analysis. The bioinformatics tools to identify and characterize deleterious mutations from sequence data are available for application and SIFT and GERP++ RS scores for seven major crops can be downloaded for use from Fu et al. [18]. Third, as genome editing to repair deleterious alleles has become technically possible for crop improvement [64,65], screening and mapping deleterious mutations of plant germplasm will play an important role in generating genomic profiles on deleterious genes for genome-wide or targeted allelic repairs (e.g., see [20,66,67]). The generated deleterious SNP VCF files in the Supplementary Materials could be part of genomic resources useful for mapping and exploring deleterious mutations for gene editing to purge oat or wheat deleterious alleles. Also, mapped deleterious alleles are functional genetic markers and could be more informative for genetic predictions of yield and fitness-related traits than other random genomic markers [7,20,68,69], enabling better genomic selection for plant breeding [19]. Thus, mutation mapping can serve as an additional breeding tool to make plant breeding more effective in purging deleterious mutations for better fitness and productivity [8,55,60], particularly in a long-term breeding program.

## 5. Conclusions

This study yielded several interesting findings on Canadian oat and wheat breeding gene pools. Many deleterious genetic variants were found in all chromosomes of oat and wheat genomes. These deleterious variants were largely harbored in a few cultivars and were involved with diverse biological processes, cellular components, and molecular functions. The estimated mutation burdens varied among the oat and wheat cultivars. These burdens were found to increase from early to recent oat and wheat cultivars and were associated with higher cultivar yields. The genetic shifts and expansions were also found from early to recent oat and wheat cultivars. These findings provided the first empirical evidence of elevated mutation burdens in Canadian oat and wheat breeding gene pools and are useful for advancing effective plant breeding programs to minimize genetic risk.

## Figures and Tables

**Table 1:** Results of annotating genetic variants detected in 141 oat and 142 wheat cultivars and identifying deleterious SNPs.

Variant	141 Oat Cultivars	142 Wheat Cultivars
***SNP calling and filtering***		
Total SNPs without missing values	253,264	270,622
***SNP annotation with VEP (most severe consequences)***		
Missense_variant (MV)	74,655	94,003
Proportion of MV in total SNPs	0.2948	0.3474
Synonymous_variant (SV)	125,064	144,071
Proportion of SV in total SNPs	0.4938	0.5324
Splice_acceptor_variant	155	279
Splice_donor_variant	137	341
Stop_gained	582	520
Stop_lost	147	84
Start_lost	57	51
Splice_region_variant	659	1214
Stop_retained_variant	184	127
Coding_sequence_variant	0	3
5_prime_UTR_variant	14,382	29,115
3_prime_UTR_variant	52,173	57,649
Non_coding_transcript_exon_variant	0	190
Intron_variant	5598	8104
Upstream_gene_variant	83,198	71,843
Downstream_gene_variant	131,064	114,488
Intergenic_variant	2937	20,233
Loss-of-function variant *		
Total count	1921	2616
Proportion	0.0076	0.0097
SIFT analysis with CT **		
SIFT-deleterious SNPs (SDS)	12,182	12,855
Proportion of SDS in total SNPs	0.0481	0.0475
Deleterious_low_confidence SNPs	4026	4664
Tolerated SNPs	152,099	57,736
Tolerated_low_confidence SNPs	NA ***	17,559
***Deleterious SNPs by SIFT+RS***		
SDS+RS-filtered SNPs (RSD)	5726	3022
Proportion of RSD in total SNPs	0.02261	0.01117
Fixed RSD	3	16
Proportion of fixed RSD in total SNPs	0.000012	0.000059
Weakly deleterious with RS < 1	2348	2834
Mildly deleterious with RS of 1–3	2295	161
Highly deleterious with RS > 3	1083	27

* Loss-of-function variants consist of those variants from the annotation classes of three “STOP_”, three “Splice_” and one “Start_lost”. ** SIFT-filtered with canonical transcripts (CT). *** NA = not available.

## Data Availability

The original RNA-Seq sequence data were deposited in NCBI’s SRA database under BioProject IDs, PRJNA1132186 and PRJNA1135233, respectively. Some research outputs can be found in the Supplementary Materials.

## Supplementary Materials

The following supporting information can be downloaded at https://doi.org/10.6084/m9.figshare.29043914 (accessed on 1 June 2025). It consists of three sections. A: supplemental tables—Tables S1–S6; B: supplemental figures—Figures S1–S9; and C: supplemental output files—oatc141-del-SNP.vcf.gz (2.3 Mb in size) and wheatc142-del-SNP.vcf.gz (1.3 Mb in size).

## Abbreviations

The following abbreviations are used in this manuscript:

AAFC	Agriculture and Agri-Food Canada
BAM	Binary alignment map
CDC	Crop Development Centre
dSNP	Deleterious simple nucleotide polymorphism
FASTA	A text-based file format used to store nucleotide and protein sequences
FASTQ	A text-based file format used to store a nucleotide sequence and its quality scores
GO	Gene ontology
PCA	Principal component analysis
PCR	Polymerase chain reaction
PGRC	Plant Gene Resources of Canada
RNA-Seq	RNA sequencing
RS	Rejected substitution
SCIC	Saskatchewan Crop Insurance Corporation
SIFT	Sorting intolerant from tolerant
SNP	Single nucleotide polymorphism
VCF	Variant call format
VEP	Variant effect predictor
