# Supplementary material for: Elevated Mutation Burdens in Canadian Oat and Wheat Cultivars Released over the Past Century
Source: Cells. 2025 Jun 4;14(11):844. doi: 10.3390/cells14110844 (PMC12155307; doi:10.3390/cells14110844)

Elevated mutation burdens in Canadian oat and wheat cultivars released over the past century

Yong-Bi Fu* and Carolee Horbach

Supplemental materials

A: Supplemental tables

Table S1. List of 141 registered Canadian oat cultivars, along with their breeding origin, year, breeding period, accession and sequencing label

Table S1a. Oat yield reports used to collect or adjust yield data for 92 oat cultivars listed in Table S1. The major report for collecting cultivar yield was the SCIC Oats Provincial Average Yields by Variety (2019-2023). The adjusted yields for the other cultivars that were not available in the major report were obtained from the other yield reports using the shared cultivar between two specific yield reports for adjustments

Table S2. List of 142 registered Canadian wheat cultivars, along with their breeding origin, year, breeding period, accession and sequencing label

Table S2a. Wheat yield reports used to collect or adjust yield data for 105 wheat cultivars listed in Table S2. The major report for collecting cultivar yield was the data published by Iqbal et al. (2016) in Crop Sci 56:613-624. The adjusted yields for the other cultivars that were not available in the major report were obtained from the other yield reports using the shared cultivar between two specific yield reports for adjustments

Table S3. Summary of sequence reads for 141 oat cultivars

Table S4. Summary of sequence reads for 142 wheat cultivars

Table S5. Estimates of three mutation burdens per deleterious locus (heterozygous, homozygous and total) and gene expression (or average transcripts per million per gene) at the early seedling stage for the 141 oat cultivars

Table S6. Estimates of three mutation burdens per deleterious locus (heterozygous, homozygous and total) and gene expression (or average transcripts per million per gene) at the early seedling stage for the 142 wheat cultivars

B. Supplemental figures

Fig. S1. The distributions of all identified SNPs (A and C) and deleterious SNPs (B and D) across 21 oat and 21 wheat chromosomes for 141 oat and 142 wheat cultivars, respectively. The identified deleterious SNPs in oat and wheat cultivars were widely distributed across their 21 chromosomes

Fig. S2. The distributions of minor allele frequencies for all SNP data (A) and deleterious SNP data (B) in 141 oat and 142 wheat cultivars. The distribution patterns are expected, except the considerable heterozygous SNP genotypes observed in the wheat cultivars (as shown in A2)

Fig. S3. The distributions of GERP++ RS scores for the identified deleterious SNPs in oat and wheat cultivars, showing the severity of their possible detrimental impacts. SNPs with RS scores <1, 1-3, or >3 would be classified as weakly, mildly, or highly deleterious, respectively. There were 1083 and 27 highly deleterious oat and wheat SNPs, respectively

Fig. S4. REVIGO gene ontology treemap showing cluster representatives of the 537 biological processes associated with significant 585 GO terms that were extracted from 7,157 genes associated with the oat deleterious SNPs

Fig. S5. REVIGO gene ontology treemap showing cluster representatives of the 232 biological processes associated with significant 262 GO terms that were extracted from 3,533 genes associated with the wheat deleterious SNPs

Fig. S6. Top 40 cellular components inferred with g:Profiler from significant GO terms of 7,157 oat (A) and 3,533 wheat (B) genes associated with the identified deleterious SNPs. The 19 cellular components shared between oat and wheat were highlighted in brown

Fig. S7. Top 40 molecular functions inferred with g:Profiler from significant GO terms of 7,157 oat (A) and 3,533 wheat (B) genes associated with the identified deleterious SNPs. The four molecular functions shared between oat and wheat were highlighted in brown

Fig. S8. The patterns of deleterious gene expressions in early seedling growth over the registration years of the 141 oat (A) and 142 wheat (B) cultivars, as measured with the mean transcripts per million per gene. When the wheat cultivar released in 1845 was excluded, the linear regression of gene expressions over the 141 wheat cultivar registration years remained not statistically significant (the coefficient=-0.0024, R^2=0.0124, P=0.10)

Fig. S9. The ranking patterns of estimated three mutation burdens per deleterious locus (total, heterozygous, homozygous) for 141 oat (A) and 142 wheat (B) cultivars

C. Supplemental output files

1. The VCF file for 141 oat cultivars with 5,726 deleterious SNPs: oatc141-del-SNP.vcf.gz (2.3Mb in size)
2. The VCF file for 142 wheat cultivars with 3,022 deleterious SNPs: wheatc142-del-SNP.vcf.gz (1.3Mb in size)

Table S1. List of 141 registered Canadian oat cultivars, along with their breeding origin, year, breeding period, accession and sequencing label

Cultivar	Program[a]	Year[b]	Period[c]	Yield[d]	CN[e]	Sequence label
CDC Endure	CDC	2019	p10	100.6	NA	001_CDC-Endure
CDC Skye	CDC	2019	p10	89.8*	NA	002_CDC-Skye
AAC Kongsore	BRDC	2018	p10	91.7*	NA	003_AAC-Kongsore
CDC Arborg	CDC	2017	p10	98.3	NA	004_CDC-ARBORG
CDC Norseman	CDC	2015	p10	94.9*	CN120098	005_CDC-NORSEMAN
AAC Richmond	ORDC	2014	p10	91.1*	CN120102	006_AAC-RICHMOND
AAC Oravena	CRC	2014	p10	35.7	NA	007_AAC-ORAVENA
AAC Oaklin	ORDC	2014	p10	86.6*	CN120103	008_AAC-OAKLIN
CDC Ruffian	CDC	2013	p10	102.3	CN116269	009_CDC-RUFFIAN
AAC Justice	CRC	2013	p10	97.4*	CN119035	010_AAC-JUSTICE
Stride	CRC	2012	p10	89.8*	CN116280	011_STRIDE
AAC Bullet	ORDC	2012	p10	106.3*	CN120100	012_AAC-BULLET
AAC Roskens	ORDC	2012	p10	NA	CN120101	013_AAC-ROSKENS
CDC Big Brown	CDC	2011	p10	89.8*	CN114469	014_CDC-BIG-BROWN
CDC Nasser	CDC	2011	p10	79.0	CN114470	015_CDC-NASSER
CDC Morrison	CDC	2011	p10	95.5	CN116268	016_CDC-MORRISON
CDC Seabiscuit	CDC	2010	p10	94.6*	CN114403	017_CDC-SEABISCUIT
Bradley	ORDC	2010	p10	93.2*	CN114404	018_BRADLEY
CDC Minstrel	CDC	2008	p9	85.1	CN113941	019_CDC-MINSTREL
Stainless	CRC	2008	p9	91.1*	CN113504	020_STAINLESS
Souris	NDAES	2008	p9	82.5	CN113938	021_SOURIS
Dieter	ORDC	2008	p9	83.5*	CN114405	022_DIETER
Oscar	ORDC	2008	p9	NA	CN114406	023_OSCAR
Gehl hulless	ORDC	2008	p9	NA	CN113508	024_GEHL
Summit	CRC	2008	p9	86.3	CN113503	025_SUMMIT
CDC Profi	CDC	2007	p9	85.5*	CN113940	026_CDC-PROFI
Robust	UOG	2006	p9	NA	CN119559	027_ROBUST
HiFi	NDAES	2006	p9	96.0*	CN119463	028_HIFI
Jordan	CRC	2006	p9	91.7*	CN119501	029_JORDAN
Domingo	LSS	2006	p9	83.7*	CN120099	030_DOMINGO
CDC SO-I	CDC	2006	p9	73.3	CN113505	031_CDC-SO-I
Bia	LSS	2006	p9	98.1*	CN119551	032_BIA
Leggett	CRC	2005	p9	68.0	CN119503	033_LEGGETT
SW Betania	LSS	2005	p9	NA	CN119572	034_SW-BETANIA
Canmore	SCRDC	2005	p9	88.2*	CN113934	035_CANMORE
Shadow hulless	I.US	2005	p9	57.5*	CN119560	036_SHADOW
Sutton	Semican	2005	p9	NA	CN119562	037_SUTTON
Lee Williams hulless	LRC	2004	p9	69.6*	CN113597	038_LEE-WILLIAMS
Sherwood	ORDC	2004	p9	85.8*	CN119561	039_SHERWOOD
Jay	UOG	2004	p9	NA	CN119464	040_JAY
CDC Sol-Fi	CDC	2004	p9	86.0	CN119479	041_CDC-SOL-FI
Alcyon	ORDC	2004	p9	NA	CN113932	042_ALCYON
Prescott	ORDC	2004	p9	NA	CN119558	043_PRESCOTT
CDC Weaver	CDC	2004	p9	90.4*	CN113595	044_CDC-WEAVER
Furlong	CRC	2003	p9	85.4*	CN113598	045_FURLONG
Manotick	ECORC	2002	p9	89.0*	CN119552	046_MANOTICK
Navan	ECORC	2002	p9	NA	CN19199	047_NAVAN
Ronald	CRC	2001	p9	84.2*	CN99035	048_RONALD
Pinnacle	CRC	2000	p9	75.4	CN99034	049_PINNACLE
CDC Bell	CDC	1998	p8	NA	CN99044	050_CDC-BELL
AC Ernie hulless	ECORC	1997	p8	75.1*	CN99046	051_AC-ERNIE
AC Rebel	CRC	1996	p8	63.4*	CN99043	052_AC-REBEL
AC Fregeau hulless	ECORC	1996	p8	NA	CN99048	053_AC-FREGEAU
AC Percy hulless	ECORC	1994	p8	55.2*	CN18136	054_AC-PERCY-HULLESS
AC Baton hulless	ECORC	1994	p8	55.3*	CN99047	055_AC-BATON
AC Preakness	CRC	1993	p8	63.5*	CN99045	056_AC-PREAKNESS
AC Hunter	ECORC	1992	p8	84.7*	CN46738	057_AC-HUNTER
AC Belmont hulless	CRC	1992	p8	61.6*	CN52130	058_AC-BELMONT
AC Stewart	ECORC	1991	p8	85.5*	CN119548	059_AC-STEWART
AC Lotta hulless	ECORC	1991	p8	59.9*	CN52129	060_AC-LOTTA
Waldern	LRC	1990	p8	79.4*	CN18133	061_WALDERN
Appalaches	SCRDC	1989	p7	NA	CN99040	062_APPALACHES
Sylva	SCRDC	1989	p7	NA	CN99041	063_SYLVA
Ultima	SCRDC	1989	p7	110.3*	CN99049	064_ULTIMA
Cluan	SCRDC	1988	p7	NA	CN99037	065_CLUAN
Newman	ECORC	1988	p7	83.2*	CN45979	066_NEWMAN
Quamby	SCRDC	1988	p7	NA	CN99038	067_QO-220-29-QUAMBY
Derby	CDC	1988	p7	62.8	CN46754	068_DERBY
Capital	SCRDC	1987	p7	85.8*	CN45129	069_CAPITAL
Robert	CRC	1987	p7	58.2*	CN99039	070_ROBERT
Nova	SCRDC	1986	p7	84.1*	CN41099	071_NOVA
Baldwin	MDC	1985	p7	75.6*	CN17843	072_BALDWIN
Tibor hulless	ECORC	1985	p7	49.4*	CN17824	073_TIBOR
Riel	CRC	1985	p7	71.7*	CN17835	074_RIEL
Marion	SCRDC	1985	p7	85.0*	CN17834	075_MARION
Jasper	LRC	1985	p7	57.7*	CN17733	076_JASPER
Calibre	CDC	1983	p7	76.6	CN42931	077_CALIBRE
Donald	ECORC	1982	p7	69.8*	CN51839	078_DONALD
Kamouraska	SCRDC	1982	p7	NA	CN42933	079_KAMOURASKA
OAC Woodstock	UOG	1982	p7	74.1*	CN43400	080_OAC-WOODSTOCK
Dumont	CRC	1982	p7	59.4*	CN42932	081_DUMONT
Fidler	CRC	1980	p7	54.8*	CN37174	082_FIDLER
Lamar	SCRDC	1979	p6	69.8*	CN39341	083_LAMAR
Cascade	LRC	1979	p6	61.5*	CN9437	084_CASCADE
Manic	SCRDC	1979	p6	NA	CN2216	085_MANIC
Sentinel	ECORC	1978	p6	66.9*	CN33965	086_SENTINEL
Foothill	ECORC	1978	p6	NA	CN1972	087_FOOTHILL
Laurent	MDC	1977	p6	54.5*	CN33979	088_LAURENT
Alma	SCRDC	1974	p6	72.8*	CN33005	089_ALMA
Elgin	UOG	1974	p6	65.1*	CN33003	090_ELGIN
Hudson	CRC	1974	p6	NA	CN33004	091_HUDSON
Scott	ECORC	1972	p6	69.3*	CN32988	093_SCOTT
Random	LRC	1971	p6	73.3*	CN1790	094_RANDOM
Fraser	ARC	1967	p5	69.9*	CN34093	095_FRASER
Grizzly	UOA	1967	p5	68.5*	CN1807	096_GRIZZLY
Kelsey	IHRS	1966	p5	NA	CN4748	097_KELSEY
Sioux	CRC	1966	p5	NA	CN5106	098_SIOUX
Harmon	CRC	1965	p5	68.5*	CN4686	099_HARMON
Russell	ECORC	1960	p5	68.8*	CN57374	100_RUSSELL
Pendek	LRC	1959	p4	NA	CN28892	101_PENDEK
Fredericton	ECORC	1957	p4	NA	CN4655	102_FREDERICTON
Fundy	ECORC	1957	p4	65.6*	CN57138	103_FUNDY
Glen	MDC	1957	p4	66.9*	CN57450	104_GLEN
Shield	ECORC	1956	p4	60.1*	CN57063	105_SHIELD
Vicar hulless	CRC	1956	p4	NA	CN57141	106_VICAR-HULLESS
Rodney	CRC	1953	p4	NA	CN56534	107_RODNEY
Scotian	ECORC	1953	p4	NA	CN57057	108_SCOTIAN
Shefford	MDC	1953	p4	NA	CN56809	109_SHEFFORD
Simcoe	UOG	1953	p4	NA	CN56639	110_SIMCOE
Torch hulless	CDC	1951	p4	NA	CN57115	111_TORCH-HULLESS
Fortune	CDC	1948	p3	NA	CN2939	112_FORTUNE
Lanark(2)	ECORC	1948	p3	NA	CN55042	113_LANARK-2
Abegweit	ECORC	1947	p3	NA	CN55041	114_ABEGWEIT
Beacon	ECORC	1947	p3	NA	CN54850	115_BEACON
Garry	CRC	1947	p3	65.4*	CN54964	116_GARRY
Beaver	ECORC	1945	p3	NA	CN54764	117_BEAVER
Larain	LRC	1945	p3	NA	CN56416	118_LARAIN
Roxton	MDC	1943	p3	53.6*	CN54539	119_ROXTON
Exeter	CRC	1942	p3	65.0*	CN54559	120_EXETER
Ajax	CRC	1941	p3	61.4*	CN54558	121_AJAX
Brighton hulless	ECORC	1941	p3	NA	CN54561	122_BRIGHTON-HULLESS
Valor	CDC	1940	p3	NA	CN54542	123_VALOR
Lanark(1)	MDC	1939	p2	NA	CN54258	124_LANARK-1
Mabel	MDC	1939	p2	NA	CN54259	125_MABEL
Eagle	I.SW	1937	p2	NA	CN54059	126_EAGLE
Erban	UOG	1937	p2	56.2*	CN54307	127_ERBAN
Bell	I.SC	1932	p2	NA	CN34079	128_BELL
Cartier	MDC	1932	p2	NA	CN53891	129_CARTIER
Vanguard	CRC	1930	p2	61.2*	CN54306	130_VANGUARD
Early Triumph	MDC	1927	p1	NA	CN53877	131_EARLY-TRIUMPH
Gold Rain	I.SW	1926	p1	63.1*	CN53714	132_GOLD-RAIN
Hajira	I.AL	1926	p1	NA	CN1954	133_HAJIRA
Gopher	I.US	1923	p1	57.4*	CN53661	134_GOPHER
Legacy	ECORC	1920	p1	NA	CN34096	135_LEGACY
Victory	I.SW	1911	p1	62.5*	CN53419	136_VICTORY
Sixty Day	I.RU	1905	p1	60.1*	CN53083	137_SIXTY-DAY
Swedish Select	I.SW	1902	p1	NA	CN53052	138_SWEDISH-SELECT
Alaska	I.US	1900	p1	26.8*	CN53097	139_ALASKA
Old Island Black	I.UN	1900	p1	NA	CN52996	140_OLD-ISLAND-BLACK
Joanette	I.SW	1889	p1	NA	CN53273	141_JOANETTE
Banner	I.US	1886	p1	65.0*	CN53004	142_BANNER

[a]The breeding program is coded here: for AAFC breeding program, ARC = Agassiz Research Centre, BRDC = Brandon Research and Development Centre, CRC = Cereal Research Centre, ECORC = Eastern Cereal and Oilseed Research Centre, IHRS = Indian Head Research Station, LRC = Lacombe Research Centre; for university program, MDC = Macdonald College, McGill University, UOG = University of Guelph, CDC = Crop Development Center, University of Saskatchewan, and UOA = University of Alberta; for industry program, APAU = AgriPro and Agricore United joint breeding program and LSS = Lantmännen Seed, Sweden; and for introduction, Algeria (I.AL), Russia (I.RU), Scotland (I.SC), Sweden (I.SW), USA (I.US), North Dakota Agricultural Experiment Station (I.NDAES), and Unknown (I.UN).

[b]Year of cultivar release or registration.

[c]The breeding period was defined following Fu et al. (2003).

[d]Yield (bu/ac) was acquired from related reference listed in Table S1a and those with stars were adjusted based on different data sources (see Table S1a for explanation).

[e]CN = Canadian National accession number in the PGRC collection. NA=not from the PGRC collection, but acquired directly from Canadian plant breeders.

Table S1a. Oat yield reports used to collect or adjust yield data for 92 oat cultivars listed in Table S1. The major report for collecting cultivar yields was the SCIC Oats Provincial Average Yields by Variety (2019-2023). The adjusted yields for the other cultivars that were not available in the major report were obtained from the other yield reports using the shared cultivar between two specific yield reports for adjustments.

Cultivar	Yield reference	Online availability (accessed 25 Feb 2025)	Adjusted using
CDC Endure	SCIC Oats Provincial Average Yields by Variety(2019-2023)	www.producer.com/digital-edition/yield-saskatchewan_2024-03-20	Not adjusted
CDC Skye	Seed Manitoba Variety Selection & Growers Source Guide(2022)	seedmb.ca/wp-content/uploads/2021/11/SMB_2022.pdf	CDC Endure
AAC Kongsore	Seed Manitoba Variety Selection & Growers Source Guide(2022)	seedmb.ca/wp-content/uploads/2021/11/SMB_2022.pdf	CDC Endure
CDC Arborg	SCIC Oats Provincial Average Yields by Variety(2019-2023)	www.producer.com/digital-edition/yield-saskatchewan_2024-03-20	Not adjusted
CDC Norseman	Seed Manitoba Variety Selection & Growers Source Guide(2022)	seedmb.ca/wp-content/uploads/2021/11/SMB_2022.pdf	CDC Endure
AAC Richmond	Can. J. Plant Sci. 97:923–927(2017)	doi.org/10.1139/cjps-2017-0050	Dieter
AAC Oravena	SCIC Oats Provincial Average Yields by Variety(2019-2023)	www.producer.com/digital-edition/yield-saskatchewan_2024-03-20	Not adjusted
AAC Oaklin	Maritime Cereal Cultivars Performance Trials(2023)	princeedwardisland.ca/sites/default/files/publications/af_cerealguide.pdf	CDC Endure
CDC Ruffian	SCIC Oats Provincial Average Yields by Variety(2019-2023)	www.producer.com/digital-edition/yield-saskatchewan_2024-03-20	Not adjusted
AAC Justice	Seed Manitoba Variety Selection & Growers Source Guide(2022)	seedmb.ca/wp-content/uploads/2021/11/SMB_2022.pdf	CDC Endure
Stride	Seed Manitoba Variety Selection & Growers Source Guide(2022)	seedmb.ca/wp-content/uploads/2021/11/SMB_2022.pdf	CDC Endure
AAC Bullet	Can. J. Plant Sci. 97:731–735(2017)	doi.org/10.1139/cjps-2016-0101	Sherwood
CDC Big Brown	Seed Manitoba Variety Selection & Growers Source Guide(2022)	seedmb.ca/wp-content/uploads/2021/11/SMB_2022.pdf	CDC Endure
CDC Nasser	SCIC Oats Provincial Average Yields by Variety(2019-2023)	www.producer.com/digital-edition/yield-saskatchewan_2024-03-20	Not adjusted
CDC Morrison	SCIC Oats Provincial Average Yields by Variety(2019-2023)	www.producer.com/digital-edition/yield-saskatchewan_2024-03-20	Not adjusted
CDC Seabiscuit	Seed Manitoba Variety Selection & Growers Source Guide(2015)	seedmb.ca/wp-content/uploads/2016/11/seed_mb_2015.pdf	CDC Ruffian
Bradley	Seed Manitoba Variety Selection & Growers Source Guide(2015)	seedmb.ca/wp-content/uploads/2016/11/seed_mb_2015.pdf	CDC Ruffian
CDC Minstrel	SCIC Oats Provincial Average Yields by Variety(2019-2023)	www.producer.com/digital-edition/yield-saskatchewan_2024-03-20	Not adjusted
Stainless	Seed Manitoba Variety Selection & Growers Source Guide(2015)	seedmb.ca/wp-content/uploads/2016/11/seed_mb_2015.pdf	CDC Ruffian
Souris	SCIC Oats Provincial Average Yields by Variety(2019-2023)	www.producer.com/digital-edition/yield-saskatchewan_2024-03-20	Not adjusted
Dieter	New Brunswick Cereal Guide(2014)	www2.gnb.ca/content/dam/gnb/Departments/10/pdf/Agriculture/FieldCrops-GrandesCultures/cereal2014.pdf	Canmore
Summit	SCIC Oats Provincial Average Yields by Variety(2019-2023)	www.producer.com/digital-edition/yield-saskatchewan_2024-03-20	Not adjusted
CDC Profi	Seed Manitoba Variety Selection & Growers Source Guide(2015)	seedmb.ca/wp-content/uploads/2016/11/seed_mb_2015.pdf	CDC Ruffian
HiFi	Seed Manitoba Variety Selection & Growers Source Guide(2015)	seedmb.ca/wp-content/uploads/2016/11/seed_mb_2015.pdf	CDC Ruffian
Jordan	Seed Manitoba Variety Selection & Growers Source Guide(2022)	seedmb.ca/wp-content/uploads/2021/11/SMB_2022.pdf	CDC Endure
Domingo	New Brunswick Cereal Guide(2014)	www2.gnb.ca/content/dam/gnb/Departments/10/pdf/Agriculture/FieldCrops-GrandesCultures/cereal2014.pdf	Canmore
CDC SO-I	SCIC Oats Provincial Average Yields by Variety(2019-2023)	www.producer.com/digital-edition/yield-saskatchewan_2024-03-20	Not adjusted
Bia	Seed Manitoba Variety Selection & Growers Source Guide(2015)	seedmb.ca/wp-content/uploads/2016/11/seed_mb_2015.pdf	CDC Ruffian
Leggett	SCIC Oats Provincial Average Yields by Variety(2019-2023)	www.producer.com/digital-edition/yield-saskatchewan_2024-03-20	Not adjusted
Canmore	Maritime Cereal Cultivars Performance Trials(2023)	princeedwardisland.ca/sites/default/files/publications/af_cerealguide.pdf	CDC Endure
Shadow hulless	Maritime Oat Registration Recommendation Test(2018)	atlanticgrainscouncil.ca/wp-content/uploads/2023/01/2019-oat-variety-work.pdf	Nova
Lee Williams	Can. J. Plant Sci. 89(4):665–669(2009)	doi.org/10.4141/CJPS08197	Ronald
Sherwood	New Brunswick Cereal Guide(2014)	www2.gnb.ca/content/dam/gnb/Departments/10/pdf/Agriculture/FieldCrops-GrandesCultures/cereal2014.pdf	Canmore
CDC Sol-Fi	SCIC Oats Provincial Average Yields by Variety(2019-2023)	www.producer.com/digital-edition/yield-saskatchewan_2024-03-20	Not adjusted
CDC Weaver	Seed Manitoba Variety Selection & Growers Source Guide(2015)	seedmb.ca/wp-content/uploads/2016/11/seed_mb_2015.pdf	CDC Ruffian
Furlong	Seed Manitoba Variety Selection & Growers Source Guide(2022)	seedmb.ca/wp-content/uploads/2021/11/SMB_2022.pdf	CDC Endure
Manotick	Can. J. Plant Sci. 97:731–735(2017)	doi.org/10.1139/cjps-2016-0101	Sherwood
Ronald	Seed Manitoba Variety Selection & Growers Source Guide(2022)	seedmb.ca/wp-content/uploads/2021/11/SMB_2022.pdf	CDC Endure
Pinnacle	SCIC Oats Provincial Average Yields by Variety(2019-2023)	www.producer.com/digital-edition/yield-saskatchewan_2024-03-20	Not adjusted
AC Ernie hulless	Progress Report - Ontario Performance Trials(1999)	cereals.gocrops.ca/wp-content/uploads/sites/3/2024/02/1999_OCCC_Progress_Report.pdf	AC Stewart
AC Rebel	Can. J. Plant Sci. 81:89–91(2001)	doi.org/10.4141/P00-034	Derby
AC Percy hulless	Can. J. Plant Sci. 73:835–837(1993)	doi.org/10.4141/cjps93-107	Tibor
AC Baton hulless	Can. J. Plant Sci. 72:443–445(1992)	doi.org/10.4141/cjps92-051	Tibor
AC Preakness	Yield Manitoba Guide(2003)	masc.mb.ca/masc.nsf/ym_2003_full_issue.pdf	Derby
AC Hunter	Can. J. Plant Sci. 73:1099–1101(1993)	doi.org/10.4141/cjps93-147	Nova
AC Belmont hulless	Can. J. Plant Sci. 89:665–669(2009)	doi.org/10.4141/CJPS08197	Ronald
AC Stewart	Can. J. Plant Sci. 72:447–449(1992)	doi.org/10.4141/cjps92-052	Nova
AC Lotta hulless	Can. J. Plant Sci. 72:443–445(1992)	doi.org/10.4141/cjps92-051	Tibor
Waldern	Can. J. Plant Sci. 71:511–513(1991)	doi.org/10.4141/cjps91-071	Calibre
Ultima	Progress Report - Ontario Performance Trials(1996)	cereals.gocrops.ca/wp-content/uploads/sites/3/2024/02/1996_OCCC_Progress_Report.pdf	AC Lotta
Newman	Can. J. Plant Sci. 72:447–449(1992)	doi.org/10.4141/cjps92-052	Nova
Derby	SCIC Oats Provincial Average Yields by Variety(2019-2023)	www.producer.com/digital-edition/yield-saskatchewan_2024-03-20	Not adjusted
Capital	Can. J. Plant Sci. 72:447–449(1992)	doi.org/10.4141/cjps92-052	Nova
Robert	Yield Manitoba Guide(2003)	masc.mb.ca/masc.nsf/ym_2003_full_issue.pdf	Derby
Nova	New Brunswick Cereal Guide(2014)	www2.gnb.ca/content/dam/gnb/Departments/10/pdf/Agriculture/FieldCrops-GrandesCultures/cereal2014.pdf	Canmore
Baldwin	Can. J. Plant Sci. 67:499–501(1987)	doi.org/10.4141/cjps87-070	Elgin
Tibor hulless	Can. J. Plant Sci. 66:403–405(1986)	doi.org/10.4141/cjps86-054	Elgin
Riel	Yield Manitoba Guide 2003(1998-2001)	masc.mb.ca/masc.nsf/ym_2003_full_issue.pdf	Derby
Marion	Can. J. Plant Sci. 72:447–449(1992)	doi.org/10.4141/cjps92-052	Nova
Jasper	Can. J. Plant Sci. 81:89–91(2001)	doi.org/10.4141/P00-034	Derby
Calibre	SCIC Oats Provincial Average Yields by Variety(2019-2023)	www.producer.com/digital-edition/yield-saskatchewan_2024-03-20	Not adjusted
Donald	Can. J. Plant Sci. 64:411–413(1984)	doi.org/10.4141/cjps84-059	Elgin
OAC Woodstock	Can. J. Plant Sci. 67:499–501(1987)	doi.org/10.4141/cjps87-070	Elgin
Dumont	Can. J. Plant Sci. 81:89-91(2001)	doi.org/10.4141/P00-034	Derby
Fidler	Yield Manitoba Guide(2003)	masc.mb.ca/masc.nsf/ym_2003_full_issue.pdf	Derby
Lamar	Can. J. Plant Sci. 64:411–413(1984)	doi.org/10.4141/cjps84-059	Elgin
Cascade	Can. J. Plant Sci. 81:89–91(2001)	doi.org/10.4141/P00-034	Derby
Sentinel	Can. J. Plant Sci. 64:411–413(1984)	doi.org/10.4141/cjps84-059	Elgin
Laurent	Can. J. Plant Sci. 59:233–235(1979)	doi.org/10.4141/cjps79-031	Garry
Alma	Can. J. Plant Sci. 55:635–636(1975)		Garry
Elgin	Can. J. Plant Sci. 54:827–828(1974)		Garry
Scott	Can. J. Plant Sci. 59:233–235(1979)	doi.org/10.4141/cjps79-031	Garry
Random	Can. J. Plant Sci. 54:617–620(1974)		Harmon
Fraser	Can. J. Plant Sci. 54:617–620(1974)		Harmon
Grizzly	Can. J. Plant Sci. 54:617–620(1974)		Harmon
Harmon	Can. J. Plant Sci. 64:765–767(1984)	doi.org/10.4141/cjps84-105	Calibre
Russell	Can. J. Plant Sci. 41:683–684(1961)		Victory
Fundy	Can. J. Plant Sci. 41:683–684(1961)		Victory
Glen	Can. J. Plant Sci. 41:683–684(1961)		Victory
Shield	Can. J. Plant Sci. 41:683–684(1961)		Victory
Garry	Can. J. Plant Sci. 41:683–684(1961)		Victory
Roxton	Sci. Agric. 25:96–106(1944)		Victory
Exeter	Sci. Agric. 25:96–106(1944)		Victory
Ajax	Sci. Agric. 25:96–106(1944)		Victory
Erban	Sci. Agric. 25:96–106(1944)		Victory
Vanguard	Sci. Agric. 25:96–106(1944)		Victory
Gold Rain	Sci. Agric. 3:125–134(1922)		Victory
Gopher	Sci. Agric. 25:96–106(1944)		Victory
Victory	Can. J. Plant Sci. 54:617–620(1974)		Harmon
Sixty Day	Sci. Agric. 3:125–134(1922)		Victory
Alaska	Sci. Agric. 3:125–134(1922)		Victory
Banner	Sci. Agric. 3:125–134(1922)		Victory

Table S2. List of 142 registered Canadian wheat cultivars, along with their breeding origin, year, breeding period, accession and sequencing label

Cultivar	Program[a]	Year[b]	Period[c]	Yield[d]	CN[e]	Sequence label	Cultivar	Program	Year	Period	Yield	CN	Sequence label
Red Fife	I.GC	1845	p1	58.7	CN 33615	031_RedFife	AC Superb	CRC	2001	p7	74.8	CN 106382	081_ACSuperb
Ladoga	I.RU	1887	p1	NA	CN 10921	032_Ladoga	Journey	SWP	2002	p7	65.3	CN 106379	073_Journey
Stanley	ECORC	1893	p1	NA	CN 33695	033_Stanley	Lovitt	SCRDC	2002	p7	59.3	CN 106395	084_Lovitt
Preston	ECORC	1895	p1	NA	CN 11327	001_Preston	CDC Imagine	CDC	2002	p7	71.5	NA	133_CDCImagine
Huron	ECORC	1900	p1	NA	CN 33664	034_Huron	BW776 (Lillian)	SCRDC	2003	p7	69.0	CN 106394	021_BW776Lillian
Percy	ECORC	1901	p1	NA	CN 33679	035_Percy	Peace	CRC	2004	p7	NA	CN 106424	026_Peace
White Fife	ECORC	1908	p1	NA	CN 12172	036_WhiteFife	Infinity	SCRDC	2004	p7	65.6	CN 113489	028_Infinity
Marquis	ECORC	1909	p1	56.2	CN 33674	037_Marquis	Harvest	CRC	2004	p7	70.0	CN 106393	085_Harvest
Prelude	ECORC	1913	p2	NA	CN 45652	038_Prelude	CDC Go	CDC	2004	p7	80.0	CN 113453	086_CDCGo
Ruby	ECORC	1917	p2	NA	CN 11902	039_Ruby	Somerset	CRC	2005	p7	69.6	CN 113484	088_Somerset
Early Triumph	REF	1918	p2	NA	CN 10013	040_EarlyTriumph	Alvena	SCRDC	2006	p7	71.8	CN 113498	022_Alvena
Kota	I.US	1921	p2	NA	CN 1798	002_Kota	CDC Alsask	CDC	2006	p7	72.7	CN 113450	087_CDCAlsask
Supreme	REF	1921	p2	NA	CN 12011	003_Supreme	Helios	SCRDC	2006	p7	69.6	CN 113481	089_Helios
Renfrew	UOA	1924	p2	NA	CN 33688	041_Renfrew	CDC Alsask	CDC	2006	p7	72.7	CN 113450	097_CDCAlsask
Broatch's Whitehead	CDC	1925	p2	NA	CN 11140	004_BroatchsWhitehead	Fieldstar	CRC	2007	p7	72.9	CN 113495	027_Fieldstar
Garnet	ECORC	1925	p2	54.9	CN 33655	042_Garnet	Waskada	CRC	2007	p7	70.6	CN 113501	029_Waskada
Red Bobs # 222	UOA	1926	p2	NA	CN 33685	043_RedBobs222	Goodeve (BW841)	SCRDC	2007	p7	63.8	NA	099_GoodeveBW841
Ceres	I.US	1928	p2	NA	CN 9774	005_Ceres	Unity	CRC	2007	p7	71.8	NA	120_Unity
Reward	ECORC	1928	p2	NA	CN 33689	044_Reward	Stettler	SCRDC	2008	p7	72.4	CN 113496	082_Stettler
Reliance	I.US	1932	p3	NA	CN 11766	006_Reliance	Carberry	SCRDC	2009	p7	69.9	CN 114434	090_Carberry
Canus	UOA	1935	p3	NA	CN 9742	045_Canus	Muchmore	SCRDC	2009	p7	75.7	CN 114430	091_Muchmore
Thatcher	I.US	1935	p3	NA	CN 33696	074_Thatcher	Shaw	CRC	2009	p7	74.2	NA	121_Shaw
Renown	CRC	1937	p3	NA	CN 11773	007_Renown	CDC Abound	CDC	2010	p8	75.5	CN 113490	030_CDCAbound
Apex	CDC	1937	p3	NA	CN 33627	046_Apex	CDC Kernen	CDC	2010	p8	76.9	CN 114425	092_CDCKernen
Coronation	CRC	1937	p3	NA	CN 9844	075_Coronation	CDC Stanley	CDC	2010	p8	77.6	CN 114423	093_CDCStanley
Regent	CRC	1939	p3	NA	CN 11442	047_Regent	CDC Thrive	CDC	2010	p8	70.2	CN 114424	094_CDCThrive
Rescue	SCRDC	1946	p3	NA	CN 45654	048_Rescue	CDC Utmost	CDC	2010	p8	70.8	CN 114422	095_CDCUtmost
Redman	CRC	1946	p3	NA	CN 11428	049_Redman	Vesper	CRC	2010	p8	69.6	CN 114432	096_Vesper
Saunders	ECORC	1947	p3	NA	CN 33693	076_Saunders	AAC Bailey	SCRDC	2011	p8	67.2*	CN 117681	023_AACBailey
Lee	I.US	1950	p4	NA	CN 10947	008_Lee	Cardale	CRC	2011	p8	77.8	NA	122_Cardale
Chinook	SCRDC	1952	p4	NA	CN 45641	077_Chinook	CDC VR Morris	CDC	2012	p8	76.8	CN 114493	024_CDCVRMorris
Selkirk	CRC	1953	p4	NA	CN 33623	050_Selkirk	AAC Redwater	CRC	2012	p8	101.0	NA	123_AACRedwater
Lake	SRF	1954	p4	NA	CN 10923	009_Lake	CDC Plentiful	CDC	2012	p8	79.8	NA	134_CDCPlentiful
Canthatch	CRC	1959	p4	NA	CN 9740	051_Canthatch	AAC Elie	SCRDC	2013	p8	91.4	CN 117684	025_AACElie
Pembina	CRC	1959	p4	NA	CN 11280	052_Pembina	AAC Brandon	SCRDC	2013	p8	85.7	NA	100_AACBrandon
Cypress	SCRDC	1962	p4	50.2*	CN 45642	053_Cypress	AAC Penhold	SCRDC	2014	p8	85.8	NA	101_AACPenhold
Park	LRC	1963	p4	63.0	CN 45651	054_Park	AAC Prevail	CRC	2014	p8	78.6	NA	124_AACPrevail
Manitou	CRC	1965	p4	53.7*	CN 11049	055_Manitou	AAC Connery	SCRDC	2015	p8	88.2	NA	102_AACConnery
Neepawa	CRC	1969	p4	60.5	CN 11189	078_Neepawa	AAC Cameron	CRC	2015	p8	95.2	NA	125_AACCameron
Canuck	SCRDC	1973	p5	54.3*	CN 9741	056_Canuck	AAC Jatharia	BRDC	2015	p8	77.0	NA	126_AACJatharia
Sinton	SCRDC	1975	p5	101.0*	CN 33728	057_Sinton	AAC Tradition	BRDC	2015	p8	74.7*	NA	127_AACTradition
Chester	LBRDC	1976	p5	54.3*	CN 45640	058_Chester	CDC Bradwell	CDC	2015	p8	70.0	NA	135_CDCBradwell
Benito	CRC	1979	p5	98.5*	CN 45639	059_Benito	AAC Redberry	SCRDC	2016	p8	82.0	NA	103_AACRedberry
Columbus	CRC	1980	p5	64.8	CN 37156	010_Columbus	AAC Viewfield	SCRDC	2016	p8	82.4	NA	104_AACViewfield
Leader	SCRDC	1981	p5	59.5*	CN 38926	011_Leader	AAC Concord	SCRDC	2016	p8	NA	NA	105_AACConcord
Katepwa	CRC	1981	p5	63.6	CN 51821	083_Katepwa	AAC Crossfield	CRC	2016	p8	87.0	NA	116_AACCrossfield
Lancer	SCRDC	1984	p5	59.5*	CN 17840	012_Lancer	AAC Entice	LBRDC	2016	p8	84.7	NA	117_AACEntice
Kenyon	CDC	1985	p5	62.6*	CN 43842	060_Kenyon	CDC Hughes	CDC	2016	p8	89.6	NA	136_CDCHughes
Conway	CDC	1986	p5	63.3*	CN 43840	061_Conway	CDC Landmark	CDC	2016	p8	89.1	NA	139_CDCLandmark
Laura	SCRDC	1986	p5	62.6	CN 44167	062_Laura	AAC Alida	SCRDC	2017	p8	75.0	NA	106_AACAlida
Roblin	CRC	1986	p5	63.2	CN 43847	079_Roblin	AAC Tisdale	SCRDC	2017	p8	78.9	NA	107_AACTisdale
Pasqua	CRC	1990	p6	NA	CN 106306	013_Pasqua	AAC Goodwin	SCRDC	2017	p8	85.8	NA	108_AACGoodwin
CDC Makwa	CDC	1990	p6	73.0*	CN 52587	080_CDCMakwa	AAC Warman	BRDC	2017	p8	74.8	NA	128_AACWarman
CDC Teal	CDC	1991	p6	73.8	CN 52585	063_CDCTeal	AAC Starbuck	SCRDC	2018	p8	96.0	NA	109_AACStarbuck
CDC Merlin	CDC	1992	p6	68.3	CN 52586	064_CDCMerlin	AAC Wheatland	SCRDC	2018	p8	90.3	NA	110_AACWheatland
AC Michael	LRC	1993	p6	62.2*	CN 52557	065_ACMichael	AAC Cirrus	SCRDC	2018	p8	NA	NA	111_AACCirrus
AC Eatonia	SCRDC	1993	p6	60.7	CN 106365	066_ACEatonia	AAC LeRoy	BRDC	2018	p8	81.3	NA	129_AACLeRoy
AC Domain	CRC	1993	p6	61.0	CN 106358	067_ACDomain	AAC Magnet	BRDC	2018	p8	78.2	NA	130_AACMagnet
Invader	APAU	1993	p6	NA	CN 106366	068_Invader	CDC Cordon CLPlus	CDC	2018	p8	78.9	NA	137_CDCCordonCLPlus
AC Cora	CRC	1994	p6	53.9*	CN 106353	069_ACCora	AAC Broadacres	SCRDC	2019	p8	84.7	NA	112_AACBroadacres
Pacific	CRC	1994	p6	NA	CN 106352	070_Pacific	AAC Russell	SCRDC	2019	p8	110.7	NA	113_AACRussell
AC Barrie	SCRDC	1994	p6	68.6	CN 106318	098_ACBarrie	CDC Ortona	CDC	2019	p8	66.3	NA	140_CDCOrtona
Prodigy	SWP	1995	p6	66.0	CN 106329	014_Prodigy	AAC Hockley	SCRDC	2020	p9	81.2	NA	114_AACHockley
AC Majestic	CRC	1995	p6	66.5*	CN 106357	071_ACMajestic	AAC Hodge	BRDC	2020	p9	114.0	NA	131_AACHodge
AC Elsa	SCRDC	1996	p6	68.0	CN 106314	015_ACElsa	AAC Redstar	BRDC	2020	p9	78.9	NA	132_AACRedstar
AC Cadillac	SCRDC	1996	p6	70.6	CN 106337	016_ACCadillac	CDC Skrush	CDC	2020	p9	106.6	NA	141_CDCSkrush
McKenzie	SWP	1997	p6	69.1	CN 106330	017_McKenzie	AAC Rimbey	SCRDC	2021	p9	81.2	NA	115_AACRimbey
AC Intrepid	SCRDC	1997	p6	72.4	CN 106338	018_ACIntrepid	AAC Westlock	LBRDC	2021	p9	83.8*	NA	118_AACWestlock
AC Splendor	CRC	1997	p6	63.2	CN 106351	072_ACSplendor	AAC Perform	LBRDC	2021	p9	95.9*	NA	119_AACPerform
AC Abbey	SCRDC	1998	p6	72.9	CN 106336	019_ACAbbey	CDC Pilar CLPlus	CDC	2021	p9	92.3	NA	142_CDCPilarCLPlus
CDC Bounty	CDC	1999	p6	66.2	CN 106376	020_CDCBounty	CDC Succession CLPlus	CDC	2021	p9	75.4	NA	143_CDCSuccessionCLPlus

[a]The wheat breeding program is coded here: for AAFC breeding program, BRDC = Brandon Research and Development Centre, CRC = Cereal Research Centre, ECORC = Eastern Cereal and Oilseed Research Centre, LBRDC = Lethbridge Research and Development Centre, LRC = Lacombe Research Centre, REF = Rosthern Experimental Farm, SCRDC = Swift Current Research and Development Centre, and SEF = Scott Experimental Farm; for university program, CDC = Crop Development Center, University of Saskatchewan, and UOA = University of Alberta; for industry program, APAU = AgriPro and Agricore United joint breeding program and SWP = Saskatchewan Wheat Pool; and for introduction, Galicia region of central Europe (I.GC), Russia (I.RU), and USA (I.US).

[b]Year of cultivar release or registration.

[c]The breeding period was defined following Fu et al. (2005).

[d]Yield (bu/ac) was acquired from related reference listed in Table S2a and those with stars were adjusted based on different data sources (see Table S2a for explanation).

[e]CN = Canadian National accession number in the PGRC collection. NA=not from the PGRC collection, but acquired directly from Canadian plant breeders.

Table S2a. Wheat yield reports used to collect or adjust yield data for 105 wheat cultivars listed in Table S2. The major report for collecting cultivar yields was the data published by Iqbal et al. (2016) in Crop Sci 56:613-624. The adjusted yields for the other cultivars that were not available in the major report were obtained from the other yield reports using the shared cultivar between two specific yield reports for adjustments

Cultivar	Yield Reference	Online Availability (accessed on 25 Feb 2025)	Yield Adjusted Using
Columbus	Crop Sci. 56: 613–624 (2016)	doi.org/10.2135/cropsci2015.06.0348	not adjusted
Leader	Can. J. Plant Sci. 68: 203–206 (1988)	doi.org/10.4141/cjps88-020	Neepawa
Lancer	Can. J. Plant Sci. 68: 203–206 (1988)	doi.org/10.4141/cjps88-020	Neepawa
Prodigy	Crop Sci. 56: 613–624 (2016)	doi.org/10.2135/cropsci2015.06.0348	not adjusted
AC Elsa	Crop Sci. 56: 613–624 (2016)	doi.org/10.2135/cropsci2015.06.0348	not adjusted
AC Cadillac	Crop Sci. 56: 613–624 (2016)	doi.org/10.2135/cropsci2015.06.0348	not adjusted
McKenzie	Crop Sci. 56: 613–624 (2016)	doi.org/10.2135/cropsci2015.06.0348	not adjusted
AC Intrepid	Crop Sci. 56: 613–624 (2016)	doi.org/10.2135/cropsci2015.06.0348	not adjusted
AC Abbey	Crop Sci. 56: 613–624 (2016)	doi.org/10.2135/cropsci2015.06.0348	not adjusted
CDC Bounty	Crop Sci. 56: 613–624 (2016)	doi.org/10.2135/cropsci2015.06.0348	not adjusted
BW776 (Lillian)	Crop Sci. 56: 613–624 (2016	doi.org/10.2135/cropsci2015.06.0348	not adjusted
Alvena	Crop Sci. 56: 613–624 (2016)	doi.org/10.2135/cropsci2015.06.0348	not adjusted
AAC Bailey	Can. J. Plant Sci. 94(1): 175–181 (2014)	doi.org/10.4141/cjps2013-252	Carberry
CDC VR Morris	SCIC Wheat-Hard Red Spring Provincial Average Yields by Variety (2018-2022)	www.producer.com/digital-edition/yield-saskatchewan/2023-04-01	CDC Go/Carberry/CDC Stanley*
AAC Elie	SCIC Wheat-Hard Red Spring Provincial Average Yields by Variety (2018-2022)	www.producer.com/digital-edition/yield-saskatchewan/2023-04-01	CDC Go/Carberry/CDC Stanley*
Fieldstar	Crop Sci. 56: 613–624 (2016)	doi.org/10.2135/cropsci2015.06.0348	not adjusted
Infinity	Crop Sci. 56: 613–624 (2016)	doi.org/10.2135/cropsci2015.06.0348	not adjusted
Waskada	Crop Sci. 56: 613–624 (2016)	doi.org/10.2135/cropsci2015.06.0348	not adjusted
CDC Abound	Crop Sci. 56: 613–624 (2016)	doi.org/10.2135/cropsci2015.06.0348	not adjusted
Red Fife	Crop Sci. 56: 613–624 (2016)	doi.org/10.2135/cropsci2015.06.0348	not adjusted
Marquis	Crop Sci. 56: 613–624 (2016)	doi.org/10.2135/cropsci2015.06.0348	not adjusted
Garnet	Crop Sci. 56: 613–624 (2016)	doi.org/10.2135/cropsci2015.06.0348	not adjusted
Cypress	Can. J. Plant Sci. 56: 975–976 (1976)		Neepawa
Park	Crop Sci. 56: 613–624 (2016)	doi.org/10.2135/cropsci2015.06.0348	not adjusted
Manitou	Can. J. Plant Sci. 56: 975–976 (1976)		Neepawa
Canuck	Can. J. Plant Sci. 56: 975–976 (1976)		Neepawa
Sinton	Can. J. Plant Sci. 68: 203–206 (1988)	doi.org/10.4141/cjps88-020	Neepawa
Chester	Can. J. Plant Sci. 56: 975–976 (1976)		Neepawa
Benito	Can. J. Plant Sci. 68: 203–206 (1988)	doi.org/10.4141/cjps88-020	Neepawa
Kenyon	Can. J. Plant Sci. 68: 203–206 (1988)	doi.org/10.4141/cjps88-020	Neepawa
Conway	Can. J. Plant Sci. 68: 203–206 (1988)	doi.org/10.4141/cjps88-020	Neepawa
Laura	Crop Sci. 56: 613–624 (2016)	doi.org/10.2135/cropsci2015.06.0348	not adjusted
CDC Teal	Crop Sci. 56: 613–624 (2016)	doi.org/10.2135/cropsci2015.06.0348	not adjusted
CDC Merlin	Crop Sci. 56: 613–624 (2016)	doi.org/10.2135/cropsci2015.06.0348	not adjusted
AC Michael	Can. J. Plant Sci. 56: 975–976 (1976)		Neepawa
AC Eatonia	Crop Sci. 56: 613–624 (2016)	doi.org/10.2135/cropsci2015.06.0348	not adjusted
AC Domain	Crop Sci. 56: 613–624 (2016)	doi.org/10.2135/cropsci2015.06.0348	not adjusted
AC Cora	Can. J. Plant Sci. 88(1): 157–160 (2008)	doi.org/10.4141/CJPS07003	Neepawa
AC Majestic	Can. J. Plant Sci. 87(4): 883–887 (2007)	doi.org/10.4141/CJPS06042	Neepawa
AC Splendor	Crop Sci. 56: 613–624 (2016)	doi.org/10.2135/cropsci2015.06.0348	not adjusted
Journey	Crop Sci. 56: 613–624 (2016)	doi.org/10.2135/cropsci2015.06.0348	not adjusted
Neepawa	Crop Sci. 56: 613–624 (2016)	doi.org/10.2135/cropsci2015.06.0348	not adjusted
Roblin	Crop Sci. 56: 613–624 (2016)	doi.org/10.2135/cropsci2015.06.0348	not adjusted
CDC Makwa	Can. J. Plant Sci. 72: 225–227 (1992)	doi.org/10.4141/cjps92-024	Neepawa
AC Superb	Crop Sci. 56: 613–624 (2016)	doi.org/10.2135/cropsci2015.06.0348	not adjusted
Stettler	Crop Sci. 56: 613–624 (2016)	doi.org/10.2135/cropsci2015.06.0348	not adjusted
Katepwa	Crop Sci. 56: 613–624 (2016)	doi.org/10.2135/cropsci2015.06.0348	not adjusted
Lovitt	Crop Sci. 56: 613–624 (2016)	doi.org/10.2135/cropsci2015.06.0348	not adjusted
Harvest	Crop Sci. 56: 613–624 (2016)	doi.org/10.2135/cropsci2015.06.0348	not adjusted
CDC Go	Crop Sci. 56: 613–624 (2016)	doi.org/10.2135/cropsci2015.06.0348	not adjusted
CDC Alsask	Crop Sci. 56: 613–624 (2016)	doi.org/10.2135/cropsci2015.06.0348	not adjusted
Somerset	Crop Sci. 56: 613–624 (2016)	doi.org/10.2135/cropsci2015.06.0348	not adjusted
Helios	Crop Sci. 56: 613–624 (2016)	doi.org/10.2135/cropsci2015.06.0348	not adjusted
Carberry	Crop Sci. 56: 613–624 (2016)	doi.org/10.2135/cropsci2015.06.0348	not adjusted
Muchmore	Crop Sci. 56: 613–624 (2016)	doi.org/10.2135/cropsci2015.06.0348	not adjusted
CDC Kernen	Crop Sci. 56: 613–624 (2016)	doi.org/10.2135/cropsci2015.06.0348	not adjusted
CDC Stanley	Crop Sci. 56: 613–624 (2016)	doi.org/10.2135/cropsci2015.06.0348	not adjusted
CDC Thrive	Crop Sci. 56: 613–624 (2016)	doi.org/10.2135/cropsci2015.06.0348	not adjusted
CDC Utmost	Crop Sci. 56: 613–624 (2016)	doi.org/10.2135/cropsci2015.06.0348	not adjusted
Vesper	Crop Sci. 56: 613–624 (2016)	doi.org/10.2135/cropsci2015.06.0348	not adjusted
CDC Alsask	Crop Sci. 56: 613–624 (2016)	doi.org/10.2135/cropsci2015.06.0348	not adjusted
AC Barrie	Crop Sci. 56: 613–624 (2016)	doi.org/10.2135/cropsci2015.06.0348	not adjusted
Goodeve (BW841)	Crop Sci. 56: 613–624 (2016)	doi.org/10.2135/cropsci2015.06.0348	not adjusted
AAC Brandon	SCIC Wheat-Hard Red Spring Provincial Average Yields by Variety (2018-2022)	www.producer.com/digital-edition/yield-saskatchewan/2023-04-01	CDC Go/Carberry/CDC Stanley*
AAC Penhold	Seed Manitoba Variety Selection & Growers Source Guide (2022)	seedmb.ca/wp-content/uploads/2021/11/SMB_2022.pdf	CDC Go/Carberry/CDC Stanley*
AAC Connery	SCIC Wheat-Hard Red Spring Provincial Average Yields by Variety (2018-2022)	www.producer.com/digital-edition/yield-saskatchewan/2023-04-01	CDC Go/Carberry/CDC Stanley*
AAC Redberry	SCIC Wheat-Hard Red Spring Provincial Average Yields by Variety (2018-2022)	www.producer.com/digital-edition/yield-saskatchewan/2023-04-01	CDC Go/Carberry/CDC Stanley*
AAC Viewfield	Seed Manitoba Variety Selection & Growers Source Guide (2022)	seedmb.ca/wp-content/uploads/2021/11/SMB_2022.pdf	CDC Go/Carberry/CDC Stanley*
AAC Alida	SCIC Wheat-Hard Red Spring Provincial Average Yields by Variety (2018-2022)	www.producer.com/digital-edition/yield-saskatchewan/2023-04-01	CDC Go/Carberry/CDC Stanley*
AAC Tisdale	SCIC Wheat-Hard Red Spring Provincial Average Yields by Variety (2018-2022)	www.producer.com/digital-edition/yield-saskatchewan/2023-04-01	CDC Go/Carberry/CDC Stanley*
AAC Goodwin	Seed Manitoba Variety Selection & Growers Source Guide (2022)	seedmb.ca/wp-content/uploads/2021/11/SMB_2022.pdf	CDC Go/Carberry/CDC Stanley*
AAC Starbuck	SCIC Wheat-Hard Red Spring Provincial Average Yields by Variety (2018-2022)	www.producer.com/digital-edition/yield-saskatchewan/2023-04-01	CDC Go/Carberry/CDC Stanley*
AAC Wheatland	SCIC Wheat-Hard Red Spring Provincial Average Yields by Variety (2018-2022)	www.producer.com/digital-edition/yield-saskatchewan/2023-04-01	CDC Go/Carberry/CDC Stanley*
AAC Broadacres	Seed Manitoba Variety Selection & Growers Source Guide (2022)	seedmb.ca/wp-content/uploads/2021/11/SMB_2022.pdf	CDC Go/Carberry/CDC Stanley*
AAC Russell	SCIC Wheat-Hard Red Spring Provincial Average Yields by Variety (2018-2022)	www.producer.com/digital-edition/yield-saskatchewan/2023-04-01	CDC Go/Carberry/CDC Stanley*
AAC Hockley	Seed Manitoba Variety Selection & Growers Source Guide (2022)	seedmb.ca/wp-content/uploads/2021/11/SMB_2022.pdf	CDC Go/Carberry/CDC Stanley*
AAC Rimbey	Seed Manitoba Variety Selection & Growers Source Guide (2022)	seedmb.ca/wp-content/uploads/2021/11/SMB_2022.pdf	CDC Go/Carberry/CDC Stanley*
AAC Crossfield	Seed Manitoba Variety Selection & Growers Source Guide (2022)	seedmb.ca/wp-content/uploads/2021/11/SMB_2022.pdf	CDC Go/Carberry/CDC Stanley*
AAC Entice	Seed Manitoba Variety Selection & Growers Source Guide (2022)	seedmb.ca/wp-content/uploads/2021/11/SMB_2022.pdf	CDC Go/Carberry/CDC Stanley*
AAC Westlock	Can. J. Plant Sci. 102: 949–955 (2022)	doi.org/10.1139/CJPS-2022-0018	Carberry
AAC Perform	Can. J. Plant Sci. 102: 285–291 (2022)	doi.org/10.1139/cjps-2021-0158	AAC Penhold
Unity	Crop Sci. 56: 613–624 (2016)		not adjusted
Shaw	Crop Sci. 56: 613–624 (2016)		not adjusted
Cardale	Seed Manitoba Variety Selection & Growers Source Guide (2022)	seedmb.ca/wp-content/uploads/2021/11/SMB_2022.pdf	CDC Go/Carberry/CDC Stanley*
AAC Redwater	SCIC Wheat-Hard Red Spring Provincial Average Yields by Variety (2018-2022)	www.producer.com/digital-edition/yield-saskatchewan/2023-04-01	CDC Go/Carberry/CDC Stanley*
AAC Prevail	SCIC Wheat-Hard Red Spring Provincial Average Yields by Variety (2018-2022)	www.producer.com/digital-edition/yield-saskatchewan/2023-04-01	CDC Go/Carberry/CDC Stanley*
AAC Cameron	SCIC Wheat-Hard Red Spring Provincial Average Yields by Variety (2018-2022)	www.producer.com/digital-edition/yield-saskatchewan/2023-04-01	CDC Go/Carberry/CDC Stanley*
AAC Jatharia	SCIC Wheat-Hard Red Spring Provincial Average Yields by Variety (2018-2022)	www.producer.com/digital-edition/yield-saskatchewan/2023-04-01	CDC Go/Carberry/CDC Stanley*
AAC Tradition	SeCan AAC Tradition Technical Bulletin (2017)	secan.com/system/files/AAC%20Tradition%20Wheat%20TB%20Jan%202017.pdf	Carberry
AAC Warman	SCIC Wheat-Hard Red Spring Provincial Average Yields by Variety (2018-2022)	www.producer.com/digital-edition/yield-saskatchewan/2023-04-01	CDC Go/Carberry/CDC Stanley*
AAC LeRoy	SCIC Wheat-Hard Red Spring Provincial Average Yields by Variety (2018-2022)	www.producer.com/digital-edition/yield-saskatchewan/2023-04-01	CDC Go/Carberry/CDC Stanley*
AAC Magnet	SCIC Wheat-Hard Red Spring Provincial Average Yields by Variety (2018-2022)	www.producer.com/digital-edition/yield-saskatchewan/2023-04-01	CDC Go/Carberry/CDC Stanley*
AAC Hodge	SCIC Wheat-Hard Red Spring Provincial Average Yields by Variety (2018-2022)	www.producer.com/digital-edition/yield-saskatchewan/2023-04-01	CDC Go/Carberry/CDC Stanley*
AAC Redstar	SeCan AAC Tradition Technical Bulletin (2017)	secan.com/system/files/AAC%20Tradition%20Wheat%20TB%20Jan%202017.pdf	CDC Go/Carberry/CDC Stanley*
CDC Imagine	Crop Sci. 56: 613–624 (2016)		not adjusted
CDC Plentiful	SCIC Wheat-Hard Red Spring Provincial Average Yields by Variety (2018-2022)	www.producer.com/digital-edition/yield-saskatchewan/2023-04-01	CDC Go/Carberry/CDC Stanley*
CDC Bradwell	SCIC Wheat-Hard Red Spring Provincial Average Yields by Variety (2018-2022)	www.producer.com/digital-edition/yield-saskatchewan/2023-04-01	CDC Go/Carberry/CDC Stanley*
CDC Hughes	SCIC Wheat-Hard Red Spring Provincial Average Yields by Variety (2018-2022)	www.producer.com/digital-edition/yield-saskatchewan/2023-04-01	CDC Go/Carberry/CDC Stanley*
CDC Cordon CLPlus	SeCan AAC Tradition Technical Bulletin (2017)	secan.com/system/files/AAC%20Tradition%20Wheat%20TB%20Jan%202017.pdf	CDC Go/Carberry/CDC Stanley*
CDC Landmark	SCIC Wheat - Hard Red Spring Provincial Average Yields by Variety (2018-2022)	www.producer.com/digital-edition/yield-saskatchewan/2023-04-01	CDC Go/Carberry/CDC Stanley*
CDC Ortona	SCIC Wheat - Hard Red Spring Provincial Average Yields by Variety (2018-2022)	www.producer.com/digital-edition/yield-saskatchewan/2023-04-01	CDC Go/Carberry/CDC Stanley*
CDC Skrush	SCIC Wheat - Hard Red Spring Provincial Average Yields by Variety (2018-2022)	www.producer.com/digital-edition/yield-saskatchewan/2023-04-01	CDC Go/Carberry/CDC Stanley*
CDC Pilar CLPlus	SCIC Wheat - Hard Red Spring Provincial Average Yields by Variety (2018-2022)	www.producer.com/digital-edition/yield-saskatchewan/2023-04-01	CDC Go/Carberry/CDC Stanley*
CDC Succession CLPlus	SCIC Wheat - Hard Red Spring Provincial Average Yields by Variety (2018-2022)	www.producer.com/digital-edition/yield-saskatchewan/2023-04-01	CDC Go/Carberry/CDC Stanley*

Table S3. Summary of sequence reads for 141 oat cultivars

Sequencing label for assayed cultivars	Original fastq paired reads	Trimmed fastq paired reads	Trimmed paired reads (%)	Total reads in BAM file	Total mapped reads in BAM file	Mapped reads (%)
1_CDC-Endure	11046886	10003284	90.55	22834500	19995014	87.56
2_CDC-Skye	14637422	13335146	91.10	30568619	26649170	87.18
3_AAC-Kongsore	20391702	18634863	91.38	42411338	37227720	87.78
4_CDC-ARBORG	11407735	10415690	91.30	23952836	20821793	86.93
5_CDC-NORSEMAN	17065929	15651214	91.71	35995574	31271478	86.88
6_AAC-RICHMOND	13129514	12063781	91.88	27648227	24112425	87.21
7_AAC-ORAVENA	12979269	11798250	90.90	27151814	23570527	86.81
8_AAC-OAKLIN	19991044	18301101	91.55	41974411	36569538	87.12
9_CDC-RUFFIAN	14187031	12981823	91.50	29716072	25946143	87.31
10_AAC-JUSTICE	16759706	15350342	91.59	35199631	30644722	87.06
11_STRIDE	21500638	19805749	92.12	45567678	39579682	86.86
12_AAC-BULLET	9461113	8605789	90.96	19712331	17195946	87.23
13_AAC-ROSKENS	18292474	16895052	92.36	38869665	33766927	86.87
14_CDC-BIG-BROWN	14102792	12964508	91.93	29648670	25904777	87.37
15_CDC-NASSER	15648778	14323802	91.53	32839856	28615189	87.14
16_CDC-MORRISON	14619052	13393786	91.62	30713075	26737411	87.06
17_CDC-SEABISCUIT	12873548	11769466	91.42	26900781	23510371	87.40
18_BRADLEY	26846221	24705978	92.03	56578686	49372737	87.26
19_CDC-MINSTREL	17570270	16223421	92.33	37251124	32416319	87.02
20_STAINLESS	9886766	9146761	92.52	20995439	18257357	86.96
21_SOURIS	14259246	13149060	92.21	30040207	26270407	87.45
22_DIETER	18684005	17232595	92.23	39381355	34436338	87.44
23_OSCAR	17333574	15953939	92.04	36598931	31887255	87.13
24_GEHL	18575775	17036758	91.71	38902300	34040160	87.50
25_SUMMIT	21371680	19473362	91.12	44365562	38896564	87.67
26_CDC-PROFI	27345837	25069160	91.67	57267250	50082173	87.45
27_ROBUST	25027926	23033040	92.03	52771873	46028787	87.22
28_HIFI	9998744	9224298	92.25	21132259	18415445	87.14
29_JORDAN	19332796	17918350	92.68	40832394	35798140	87.67
30_DOMINGO	18118903	16723641	92.30	38387978	33430879	87.09
31_CDC-SO-I	24266040	22334361	92.04	51152851	44612466	87.21
32_BIA	14887311	13712420	92.11	31387829	27403327	87.31
33_LEGGETT	18177446	16845785	92.67	38593513	33658110	87.21
34_SW-BETANIA	21235620	19519740	91.92	44853863	39018118	86.99
35_CANMORE	26171086	24074903	91.99	54641427	48110370	88.05
36_SHADOW	10932366	10070542	92.12	23086890	20125441	87.17
37_SUTTON	15851513	14577609	91.96	33541785	29131868	86.85
38_LEE-WILLIAMS	24923288	22953654	92.10	52402234	45854546	87.50
39_SHERWOOD	18282400	16834117	92.08	38340526	33639843	87.74
40_JAY	18470614	16982032	91.94	38847138	33932287	87.35
41_CDC-SOL-FI	14421428	13239597	91.81	30276501	26438253	87.32
42_ALCYON	18061976	16650620	92.19	38076359	33276800	87.39
43_PRESCOTT	17868673	16540752	92.57	37803933	33055834	87.44
44_CDC-WEAVER	9121992	8465334	92.80	19386549	16910437	87.23
45_FURLONG	16401480	15086073	91.98	34618570	30142221	87.07
46_MANOTICK	17505514	16206335	92.58	37335111	32387579	86.75
47_NAVAN	15453111	14262273	92.29	32706377	28499643	87.14
48_RONALD	28724571	25537296	88.90	58078766	51025161	87.86
49_PINNACLE	31016100	28704958	92.55	65581956	57338834	87.43
50_CDC-BELL	30322448	27481006	90.63	62919550	54928172	87.30
51_AC-ERNIE	24927093	22949915	92.07	52449682	45874938	87.46
52_AC-REBEL	10467362	9785351	93.48	22313830	19543386	87.58
53_AC-FREGEAU	14832843	13221513	89.14	30180038	26421072	87.54
54_AC-PERCY-HULLESS	18798697	17312708	92.10	39756114	34600319	87.03
55_AC-BATON	22534060	20360843	90.36	46515427	40678222	87.45
56_AC-PREAKNESS	23214682	21022804	90.56	47849756	41975514	87.72
57_AC-HUNTER	20181690	18425146	91.30	42143374	36812302	87.35
58_AC-BELMONT	25951846	23602290	90.95	53999577	47155933	87.33
59_AC-STEWART	18573001	17130222	92.23	39339802	34234592	87.02
60_AC-LOTTA	13507838	12533539	92.79	28709722	25045788	87.24
61_WALDERN	14034807	12955114	92.31	29694710	25892343	87.20
62_APPALACHES	27526335	25682642	93.30	59000848	51322650	86.99
63_SYLVA	27126504	24854936	91.63	56711520	49674070	87.59
64_ULTIMA	22805263	21023437	92.19	47685580	42000095	88.08
65_CLUAN	29419699	27230808	92.56	62076861	54397642	87.63
66_NEWMAN	31413290	28848352	91.83	66021708	57655318	87.33
67_QO-220.29-QUAMBY	15123608	13879586	91.77	31726262	27732158	87.41
68_DERBY	12113360	11254200	92.91	25696813	22495276	87.54
69_CAPITAL	18896820	17445138	92.32	39678585	34863845	87.87
70_ROBERT	27428795	25423188	92.69	58135019	50786797	87.36
71_NOVA	20042162	18363183	91.62	41585848	36700384	88.25
72_BALDWIN	21386942	19436128	90.88	44451844	38841646	87.38
73_TIBOR	28867348	26381133	91.39	60291406	52725019	87.45
74_RIEL	22352896	20437645	91.43	46324825	40840623	88.16
75_MARION	20587146	18916630	91.89	43304664	37808854	87.31
76_JASPER	9280981	8516004	91.76	19339420	17008668	87.95
77_CALIBRE	20071438	18311822	91.23	41659374	36596629	87.85
78_DONALD	28837812	26457755	91.75	60730626	52880085	87.07
79_KAMOURASKA	18602377	17047847	91.64	38931560	34076325	87.53
80_OAC-WOODSTOCK	18601015	16960673	91.18	39059655	33891378	86.77
81_DUMONT	19153354	17492924	91.33	39871106	34939958	87.63
82_FIDLER	38371668	34702046	90.44	79278860	69347701	87.47
83_LAMAR	21157150	19366084	91.53	44088588	38677313	87.73
84_CASCADE	10069221	9133925	90.71	20921386	18254568	87.25
85_MANIC	26565620	24300244	91.47	55556773	48562766	87.41
86_SENTINEL	19562950	18068570	92.36	41309518	36115667	87.43
87_FOOTHILL	15483887	14132467	91.27	32136398	28249341	87.90
88_LAURENT	16573013	15094496	91.08	34466843	30167518	87.53
89_ALMA	19857817	18198877	91.65	41530014	36373286	87.58
90_ELGIN	30314535	27736359	91.50	63332808	55433851	87.53
91_HUDSON	27015331	24790093	91.76	56748078	49539673	87.30
93_SCOTT	17194284	15680965	91.20	35841728	31337109	87.43
94_RANDOM	20764446	18933685	91.18	43324977	37840505	87.34
95_FRASER	31306766	28762395	91.87	65933745	57477202	87.17
96_GRIZZLY	19840121	18119018	91.33	41364708	36209393	87.54
97_KELSEY	20745092	19241836	92.75	44118341	38446889	87.14
98_SIOUX	20566256	18876899	91.79	43026608	37727957	87.69
99_HARMON	30704676	28197720	91.84	64589364	56348706	87.24
100_RUSSELL	21328201	19676973	92.26	45188321	39314625	87.00
101_PENDEK	9794862	8910103	90.97	20345935	17807084	87.52
102_FREDERICTON	22730138	20695854	91.05	47210384	41363303	87.61
103_FUNDY	19115868	17459329	91.33	40076112	34898692	87.08
104_GLEN	22178032	20318494	91.62	46560989	40609226	87.22
105_SHIELD	21918262	20190908	92.12	46253368	40351566	87.24
106_VICAR-HULLESS	18071573	16565776	91.67	37783988	33101689	87.61
107_RODNEY	39397663	35948391	91.24	82100553	71831798	87.49
108_SCOTIAN	26984578	24747485	91.71	56385564	49449048	87.70
109_SHEFFORD	13559802	12436959	91.72	28519739	24852594	87.14
110_SIMCOE	23966188	21896781	91.37	50207768	43767922	87.17
111_TORCH-HULLESS	18683700	17030446	91.15	38775583	34009679	87.71
112_FORTUNE	32224336	29504910	91.56	67333427	58928169	87.52
113_LANARK-2	19569743	17821525	91.07	40755611	35623081	87.41
114_ABEGWEIT	19139987	17567700	91.79	40202265	35106734	87.33
115_BEACON	26020575	23658747	90.92	54221858	47288480	87.21
116_GARRY	31463816	28872606	91.76	66212538	57693972	87.13
117_BEAVER	18930214	17314539	91.47	39524751	34594404	87.53
118_LARAIN	20395545	18665188	91.52	42492347	37272985	87.72
119_ROXTON	22908449	21056474	91.92	48028230	42075540	87.61
120_EXETER	17437267	15914199	91.27	36348159	31801574	87.49
121_AJAX	17661872	15991077	90.54	36471178	31960222	87.63
122_BRIGHTON-HULLESS	21364060	19473900	91.15	44414003	38917271	87.62
123_VALOR	46182642	42654604	92.36	97576075	85248296	87.37
124_LANARK-1	19639998	18034836	91.83	41256040	36049253	87.38
125_MABEL	16579727	15090644	91.02	34524372	30150469	87.33
126_EAGLE	32342602	29418886	90.96	67233828	58803308	87.46
127_ERBAN	24309359	22522870	92.65	51627156	45007864	87.18
128_BELL	16678138	15289744	91.68	34816319	30548498	87.74
129_CARTIER	12854928	11707129	91.07	26656843	23400371	87.78
130_VANGUARD	19428640	17840829	91.83	40671636	35633278	87.61
131_EARLY-TRIUMPH	33579815	30542219	90.95	69739327	61049168	87.54
132_GOLD-RAIN	28325056	26127577	92.24	59477693	52200749	87.77
133_HAJIRA	21485365	19667702	91.54	44917121	39310641	87.52
134_GOPHER	37183118	34356421	92.40	78692141	68653006	87.24
135_LEGACY	27994762	25912391	92.56	58828177	51778341	88.02
136_VICTORY	16337686	14916330	91.30	34117683	29817263	87.40
137_SIXTY-DAY	18651785	16858529	90.39	38577738	33692393	87.34
138_SWEDISH-SELECT	21832506	19839524	90.87	44998591	39638848	88.09
139_ALASKA	34232097	30668500	89.59	68382814	61254190	89.58
140_OLD-ISLAND-BLACK	22639055	20907105	92.35	47797969	41776234	87.40
141_JOANETTE	13736435	12578591	91.57	28743744	25134706	87.44
142_BANNER	26291256	23952921	91.11	54750639	47879835	87.45

Table S4. Summary of sequence reads for 142 wheat cultivars

Sequencing label for assayed cultivars	Original fastq paired reads	Trimmed fastq paired reads	Trimmed paired reads	Total reads in BAM file	Total mapped reads in BAM file	Mapped reads (%)
001_Preston	22161071	21113819	95.27	49165962	41464962	84.34
002_Kota	20392569	19309126	94.69	44904405	37858113	84.31
003_Supreme	20910284	19928664	95.31	46316330	39212424	84.66
004_BroatchsWhitehead	22142061	21129531	95.43	49174522	41560988	84.52
005_Ceres	22845789	21823401	95.52	50720439	42979777	84.74
006_Reliance	18828496	17960334	95.39	41709156	35334515	84.72
007_Renown	23530208	22510450	95.67	52320400	44139422	84.36
008_Lee	23510641	22525974	95.81	52238798	44312555	84.83
009_Lake	22162922	21129948	95.34	49142919	41574375	84.60
010_Columbus	20491278	19669074	95.99	45902857	38694752	84.30
011_Leader	20930929	20063287	95.85	46680757	39458804	84.53
012_Lancer	19642420	18812963	95.78	43865285	36991482	84.33
013_Pasqua	19310685	18437604	95.48	42783077	36242432	84.71
014_Prodigy	22917837	22008665	96.03	51371257	43353923	84.39
015_ACElsa	20189844	19342265	95.80	45059481	38079327	84.51
016_ACCadillac	19686968	18826100	95.63	43831271	37103705	84.65
017_McKenzie	23934737	22769010	95.13	52741966	44823056	84.99
018_ACIntrepid	25574623	24358689	95.25	56459286	47971864	84.97
019_ACAbbey	28057171	26694917	95.14	61965025	52503023	84.73
020_CDCBounty	28365274	26966138	95.07	62296329	53045547	85.15
021_BW776Lillian	25895411	24540902	94.77	56809977	48281883	84.99
022_Alvena	24686163	23445101	94.97	54255468	46180194	85.12
023_AACBailey	27038269	25398273	93.93	58854689	49892606	84.77
024_CDCVRMorris	25194432	24061909	95.50	55649255	47404956	85.19
025_AACElie	25782821	24514191	95.08	56770710	48279231	85.04
026_Peace	27180920	25882501	95.22	59969516	51039716	85.11
027_Fieldstar	28069696	26669656	95.01	61632431	52551305	85.27
028_Infinity	23436360	22247318	94.93	51512622	43850014	85.12
029_Waskada	25025574	23831548	95.23	55180002	46904552	85.00
030_CDCAbound	22118800	21061365	95.22	48812317	41465955	84.95
031_RedFife	26229665	24944722	95.10	57738018	49030451	84.92
032_Ladoga	24080846	22999313	95.51	53256502	45215728	84.90
033_Stanley	27855501	26634602	95.62	61778978	52357243	84.75
034_Huron	25338701	23289650	91.91	53963502	45823689	84.92
035_Percy	26285054	25097307	95.48	58086554	49425920	85.09
036_WhiteFife	25257942	24074813	95.32	55657813	47379461	85.13
037_Marquis	26654346	25447805	95.47	59064885	50170084	84.94
038_Prelude	26454918	25084520	94.82	58065772	49410891	85.09
039_Ruby	26314507	25204226	95.78	58506884	49654354	84.87
040_EarlyTriumph	26216704	24959381	95.20	57999704	49196376	84.82
041_Renfrew	24685075	23369883	94.67	54203210	45988325	84.84
042_Garnet	26501878	25213251	95.14	58451926	49639153	84.92
043_RedBobs222	27903897	26613386	95.38	61873945	52413136	84.71
044_Reward	25075128	23843310	95.09	55404493	46957617	84.75
045_Canus	25967858	24736791	95.26	57444973	48715715	84.80
046_Apex	24789854	23511635	94.84	54747916	46371091	84.70
047_Regent	24343387	23217032	95.37	54021404	45744108	84.68
048_Rescue	24838805	23582089	94.94	54849325	46292534	84.40
049_Redman	22066651	19211538	87.06	44491786	37837510	85.04
050_Selkirk	25218199	20638711	81.84	47819555	40698708	85.11
051_Canthatch	21848488	18748327	85.81	43429676	36999946	85.20
052_Pembina	22432651	20073518	89.48	46431230	39552028	85.18
053_Cypress	21619845	19992634	92.47	46271180	39405105	85.16
054_Park	23289742	12180654	52.30	28231669	23995054	84.99
055_Manitou	20331636	18220538	89.62	42118236	35881240	85.19
056_Canuck	21732831	19967715	91.88	46184880	39366754	85.24
057_Sinton	24203069	23048316	95.23	53474023	45366646	84.84
058_Chester	23916396	22697206	94.90	52622654	44799538	85.13
059_Benito	22984839	21820231	94.93	50480458	43027081	85.24
060_Kenyon	22893181	21736741	94.95	50255256	42897027	85.36
061_Conway	21281414	20201570	94.93	46569916	39974908	85.84
062_Laura	20587288	19521166	94.82	45118835	38533619	85.40
063_CDCTeal	22238861	20981231	94.34	48533678	41422735	85.35
064_CDCMerlin	20217636	19145849	94.70	44094597	37825338	85.78
065_ACMichael	23199796	20977981	90.42	48397077	41269465	85.27
066_ACEatonia	21500842	20170412	93.81	46678652	39772228	85.20
067_ACDomain	21867142	20498000	93.74	47418300	40416017	85.23
068_Invader	23313731	20668938	88.66	47946310	40732204	84.95
069_ACCora	22562059	21507470	95.33	49816605	42418520	85.15
070_Pacific	20401688	18874489	92.51	43758860	37192502	84.99
071_ACMajestic	21888982	20814503	95.09	48186643	41032541	85.15
072_ACSplendor	20451777	19587017	95.77	45181618	38645023	85.53
073_Journey	19362409	18273695	94.38	42383335	36102889	85.18
074_Thatcher	20720923	19419415	93.72	44999474	38287423	85.08
075_Coronation	21324879	20203917	94.74	46775236	39906870	85.32
076_Saunders	20373905	19383007	95.14	44864201	38251519	85.26
077_Chinook	20619748	19772290	95.89	45913326	38981974	84.90
078_Neepawa	21301217	20426798	95.89	47250078	40295721	85.28
079_Roblin	19734956	18977087	96.16	44033336	37429889	85.00
080_CDCMakwa	18220645	17453784	95.79	40252514	34416271	85.50
081_ACSuperb	25938754	24544210	94.62	56760826	46976287	82.76
082_Stettler	28049772	26590271	94.80	61293796	51186496	83.51
083_Katepwa	26871301	25604852	95.29	58919761	49353646	83.76
084_Lovitt	27633854	26380828	95.47	61041380	51740584	84.76
085_Harvest	24845261	23660261	95.23	54440752	46246255	84.95
086_CDCGo	25304852	24291840	96.00	56306847	47231137	83.88
087_CDCAlsask	28113362	26988312	96.00	62424745	52395140	83.93
088_Somerset	28079327	26982462	96.09	62193417	51914158	83.47
089_Helios	27613395	26399138	95.60	60842955	50195321	82.50
090_Carberry	24529179	23429982	95.52	54224112	45663824	84.21
091_Muchmore	28185735	27010233	95.83	62332275	51598276	82.78
092_CDCKernen	24638960	23468916	95.25	54408032	46113677	84.76
093_CDCStanley	27853879	26639511	95.64	61637685	52105996	84.54
094_CDCThrive	26779656	25660806	95.82	59231054	48737095	82.28
095_CDCUtmost	27257547	26113275	95.80	60595098	50720356	83.70
096_Vesper	28884616	27714058	95.95	64181262	53483419	83.33
097_CDCAlsask	30103665	28685538	95.29	66212325	55677125	84.09
098_ACBarrie	28046606	26533179	94.60	60977428	52073977	85.40
099_GoodeveBW841	28318977	26864321	94.86	61815933	52450855	84.85
100_AACBrandon	27850583	26563966	95.38	61307744	52069302	84.93
101_AACPenhold	28732468	27384298	95.31	63448482	53808862	84.81
102_AACConnery	27862670	26569385	95.36	61277486	51692536	84.36
103_AACRedberry	26638694	25434805	95.48	59051807	49973673	84.63
104_AACViewfield	27909243	26715594	95.72	61291456	49512949	80.78
105_AACConcord	27527741	26024928	94.54	60245419	51021125	84.69
106_AACAlida	28589193	27132647	94.91	62576327	52089285	83.24
107_AACTisdale	26433617	25196737	95.32	58363130	49289045	84.45
108_AACGoodwin	27413281	26215036	95.63	60781911	51431380	84.62
109_AACStarbuck	29559892	28151960	95.24	65166492	55364327	84.96
110_AACWheatland	29518535	28194778	95.52	65194113	55277908	84.79
111_AACCirrus	27625491	26422403	95.65	60997808	50701733	83.12
112_AACBroadacres	26630734	25377498	95.29	58660798	48316982	82.37
113_AACRussell	29768885	28447588	95.56	65789853	55954249	85.05
114_AACHockley	29896228	28607917	95.69	66127187	56356111	85.22
115_AACRimbey	29722949	28490626	95.85	65907896	56085211	85.10
116_AACCrossfield	31574853	30330192	96.06	70048283	59695262	85.22
117_AACEntice	27495698	26391439	95.98	60971240	51942169	85.19
118_AACWestlock	31179263	29948950	96.05	69269941	59017296	85.20
119_AACPerform	27930167	26801818	95.96	62063848	52624674	84.79
120_Unity	29086303	27924246	96.00	64568822	54964129	85.12
121_Shaw	27540838	25682085	93.25	59528434	50425413	84.71
122_Cardale	25932507	23835585	91.91	55085156	46822537	85.00
123_AACRedwater	27345510	25871896	94.61	59849118	50877833	85.01
124_AACPrevail	25846857	23700440	91.70	54827973	46538642	84.88
125_AACCameron	28477461	27076157	95.08	62862738	53360504	84.88
126_AACJatharia	26670836	25444122	95.40	58936519	50046379	84.92
127_AACTradition	26729967	25361848	94.88	58740654	50002862	85.12
128_AACWarman	26608609	25321743	95.16	58581730	49904830	85.19
129_AACLeRoy	27399445	25754766	94.00	59560326	50728657	85.17
130_AACMagnet	25713270	24185659	94.06	56068474	47577926	84.86
131_AACHodge	25704532	24273499	94.43	56307202	47867579	85.01
132_AACRedstar	25955931	24377339	93.92	56307091	47965760	85.19
133_CDCImagine	28646933	27108841	94.63	62772082	53309244	84.93
134_CDCPlentiful	25504990	24301079	95.28	56390103	47862917	84.88
135_CDCBradwell	25444205	24145428	94.90	55828336	47506998	85.09
136_CDCHughes	24499538	23278812	95.02	53959406	45793989	84.87
137_CDCCordonCLPlus	26680054	25008528	93.73	57922964	49331835	85.17
139_CDCLandmark	24214674	23122730	95.49	53586173	45513425	84.94
140_CDCOrtona	30236914	28766866	95.14	66761647	56726613	84.97
141_CDCSkrush	27870805	26568415	95.33	61531072	52283593	84.97
142_CDCPilarCLPlus	27451290	26245545	95.61	60759408	51664808	85.03
143_CDCSuccessionCLPlus	24808209	23593365	95.10	54590339	46434166	85.06

Table S5. Estimates of three mutation burdens per deleterious locus (heterozygous, homozygous and total) and gene expression (or average transcripts per million per gene) at the early seedling stage for the 141 oat cultivars

	Mutation burden			Gene		Mutation burden			Gene
**Sequence sample label**	**Heterozygous**	**Homozygous**	**Total**	**expression**	**Sequence sample label**	**Heterozygous**	**Homozygous**	**Total**	**expression**
001_CDC-Endure	0.0234	0.0386	0.0620	5.5151	072_BALDWIN	0.0267	0.0491	0.0758	5.1083
002_CDC-Skye	0.0312	0.0575	0.0886	5.0756	073_TIBOR	0.0311	0.0613	0.0924	5.2115
003_AAC-Kongsore	0.0367	0.0775	0.1142	6.1400	074_RIEL	0.0370	0.0592	0.0962	5.5462
004_CDC-ARBORG	0.0206	0.0267	0.0473	5.3494	075_MARION	0.0266	0.0590	0.0857	5.5341
005_CDC-NORSEMAN	0.0236	0.0356	0.0592	5.3852	076_JASPER	0.0259	0.0393	0.0652	6.0546
006_AAC-RICHMOND	0.0265	0.0494	0.0759	5.5816	077_CALIBRE	0.0286	0.0531	0.0817	5.5164
007_AAC-ORAVENA	0.0331	0.0650	0.0981	5.3809	078_DONALD	0.0272	0.0646	0.0919	5.0480
008_AAC-OAKLIN	0.0381	0.0849	0.1229	5.6561	079_KAMOURASKA	0.0203	0.0470	0.0673	5.7124
009_CDC-RUFFIAN	0.0322	0.0510	0.0832	5.4833	080_OAC-WOODSTOCK	0.0300	0.0601	0.0901	5.0397
010_AAC-JUSTICE	0.0355	0.0674	0.1029	5.5003	081_DUMONT	0.0356	0.0721	0.1078	5.4866
011_STRIDE	0.0286	0.0583	0.0870	5.3183	082_FIDLER	0.0321	0.0625	0.0947	5.1148
012_AAC-BULLET	0.0453	0.0693	0.1147	5.9497	083_LAMAR	0.0240	0.0491	0.0731	5.0796
013_AAC-ROSKENS	0.0389	0.0702	0.1092	5.5341	084_CASCADE	0.0285	0.0582	0.0866	5.7554
014_CDC-BIG-BROWN	0.0279	0.0510	0.0789	5.5434	085_MANIC	0.0229	0.0515	0.0744	5.1860
015_CDC-NASSER	0.0423	0.0788	0.1210	5.9091	086_SENTINEL	0.0239	0.0442	0.0681	5.4723
016_CDC-MORRISON	0.0377	0.0681	0.1058	5.5796	087_FOOTHILL	0.0217	0.0362	0.0579	4.9683
017_CDC-SEABISCUIT	0.0313	0.0506	0.0819	5.5683	088_LAURENT	0.0220	0.0430	0.0650	5.2525
018_BRADLEY	0.0328	0.0777	0.1105	5.4822	089_ALMA	0.0286	0.0629	0.0914	5.4110
019_CDC-MINSTREL	0.0337	0.0629	0.0966	5.7277	090_ELGIN	0.0296	0.0569	0.0865	5.3146
020_STAINLESS	0.0452	0.0733	0.1186	6.4431	091_HUDSON	0.0285	0.0576	0.0861	5.5596
021_SOURIS	0.0366	0.0707	0.1073	5.6994	093_SCOTT	0.0268	0.0531	0.0799	5.4346
022_DIETER	0.0309	0.0623	0.0933	5.5633	094_RANDOM	0.0250	0.0515	0.0765	5.1586
023_OSCAR	0.0328	0.0534	0.0863	5.6563	095_FRASER	0.0227	0.0433	0.0660	5.3466
024_GEHL	0.0372	0.0676	0.1048	4.9170	096_GRIZZLY	0.0283	0.0615	0.0898	5.3906
025_SUMMIT	0.0388	0.0672	0.1060	5.3088	097_KELSEY	0.0327	0.0683	0.1009	5.3613
026_CDC-PROFI	0.0357	0.0777	0.1134	5.3920	098_SIOUX	0.0213	0.0410	0.0623	5.4204
027_ROBUST	0.0399	0.0733	0.1133	5.3559	099_HARMON	0.0313	0.0582	0.0894	5.4636
028_HIFI	0.0408	0.0685	0.1092	6.0170	100_RUSSELL	0.0254	0.0522	0.0776	5.1130
029_JORDAN	0.0371	0.0707	0.1078	6.0059	101_PENDEK	0.0286	0.0519	0.0805	5.4796
030_DOMINGO	0.0208	0.0330	0.0538	5.3009	102_FREDERICTON	0.0207	0.0355	0.0561	5.2111
031_CDC-SO-I	0.0397	0.0903	0.1300	5.4476	103_FUNDY	0.0207	0.0349	0.0556	5.0668
032_BIA	0.0227	0.0318	0.0545	5.3188	104_GLEN	0.0231	0.0459	0.0690	5.2166
033_LEGGETT	0.0280	0.0550	0.0830	5.4385	105_SHIELD	0.0255	0.0550	0.0805	5.4557
034_SW-BETANIA	0.0251	0.0444	0.0695	5.1160	106_VICAR-HULLESS	0.0250	0.0449	0.0699	5.1646
035_CANMORE	0.0286	0.0573	0.0859	5.5971	107_RODNEY	0.0306	0.0583	0.0889	5.0894
036_SHADOW	0.0354	0.0423	0.0776	5.6156	108_SCOTIAN	0.0217	0.0423	0.0639	5.2479
037_SUTTON	0.0282	0.0555	0.0837	5.3448	109_SHEFFORD	0.0442	0.0629	0.1071	5.7010
038_LEE-WILLIAMS	0.0375	0.0782	0.1158	5.4519	110_SIMCOE	0.0188	0.0356	0.0544	5.1933
039_SHERWOOD	0.0296	0.0599	0.0895	5.6188	111_TORCH-HULLESS	0.0359	0.0634	0.0993	5.3185
040_JAY	0.0391	0.0709	0.1100	5.0724	112_FORTUNE	0.0284	0.0566	0.0850	5.3246
041_CDC-SOL-FI	0.0376	0.0657	0.1033	5.6316	113_LANARK-2	0.0234	0.0515	0.0749	5.1241
042_ALCYON	0.0321	0.0597	0.0919	5.4191	114_ABEGWEIT	0.0249	0.0398	0.0647	4.9674
043_PRESCOTT	0.0304	0.0639	0.0943	5.2830	115_BEACON	0.0244	0.0482	0.0726	5.2964
044_CDC-WEAVER	0.0381	0.0555	0.0936	5.9982	116_GARRY	0.0295	0.0552	0.0847	4.9856
045_FURLONG	0.0423	0.0870	0.1292	5.6322	117_BEAVER	0.0224	0.0402	0.0625	5.5814
046_MANOTICK	0.0358	0.0713	0.1071	5.3450	118_LARAIN	0.0305	0.0527	0.0832	5.2622
047_NAVAN	0.0346	0.0653	0.0999	5.7587	119_ROXTON	0.0245	0.0597	0.0843	5.4359
048_RONALD	0.0368	0.0735	0.1103	5.3833	120_EXETER	0.0209	0.0391	0.0600	5.1747
049_PINNACLE	0.0362	0.0763	0.1125	5.3967	121_AJAX	0.0198	0.0335	0.0534	5.1834
050_CDC-BELL	0.0289	0.0445	0.0734	5.6021	122_BRIGHTON-HULLESS	0.0266	0.0449	0.0715	5.1702
051_AC-ERNIE	0.0284	0.0554	0.0837	5.3448	123_VALOR	0.0297	0.0479	0.0775	4.8822
052_AC-REBEL	0.0341	0.0632	0.0974	6.0444	124_LANARK-1	0.0173	0.0260	0.0433	5.7036
053_AC-FREGEAU	0.0328	0.0566	0.0894	5.2274	125_MABEL	0.0284	0.0531	0.0815	5.3366
054_AC-PERCY-HULLESS	0.0302	0.0597	0.0899	5.0719	126_EAGLE	0.0155	0.0246	0.0402	5.2640
055_AC-BATON	0.0367	0.0754	0.1121	4.9962	127_ERBAN	0.0241	0.0512	0.0753	5.2853
056_AC-PREAKNESS	0.0359	0.0676	0.1035	5.2669	128_BELL	0.0452	0.0772	0.1224	5.7632
057_AC-HUNTER	0.0273	0.0655	0.0928	5.7918	129_CARTIER	0.0202	0.0300	0.0502	5.0448
058_AC-BELMONT	0.0413	0.0636	0.1049	5.5559	130_VANGUARD	0.0199	0.0293	0.0492	5.4238
059_AC-STEWART	0.0372	0.0713	0.1085	5.4501	131_EARLY-TRIUMPH	0.0204	0.0362	0.0566	5.1447
060_AC-LOTTA	0.0296	0.0580	0.0876	5.4334	132_GOLD-RAIN	0.0204	0.0274	0.0479	4.8064
061_WALDERN	0.0266	0.0603	0.0869	5.4797	133_HAJIRA	0.0444	0.0236	0.0679	5.2063
062_APPALACHES	0.0290	0.0594	0.0884	5.1291	134_GOPHER	0.0208	0.0445	0.0653	4.9000
063_SYLVA	0.0300	0.0573	0.0873	5.3121	135_LEGACY	0.0183	0.0335	0.0519	5.0486
064_ULTIMA	0.0341	0.0651	0.0993	6.2019	136_VICTORY	0.0199	0.0253	0.0452	4.8712
065_CLUAN	0.0343	0.0819	0.1162	5.2947	137_SIXTY-DAY	0.0237	0.0437	0.0673	5.0730
066_NEWMAN	0.0347	0.0653	0.1000	5.3993	138_SWEDISH-SELECT	0.0214	0.0339	0.0553	4.9617
067_QO-220-29-QUAMBY	0.0404	0.0641	0.1045	5.9363	139_ALASKA	0.0389	0.0555	0.0944	5.4082
068_DERBY	0.0258	0.0503	0.0761	5.6333	140_OLD-ISLAND-BLACK	0.0339	0.0777	0.1116	5.2028
069_CAPITAL	0.0265	0.0589	0.0854	5.2395	141_JOANETTE	0.0373	0.0653	0.1026	5.5745
070_ROBERT	0.0445	0.0948	0.1394	5.6488	142_BANNER	0.0238	0.0472	0.0710	5.2754
071_NOVA	0.0272	0.0463	0.0734	5.1223					

Table S6. Estimates of three mutation burdens per deleterious locus (heterozygous, homozygous and total) and gene expression (or average transcripts per million per gene) at the early seedling stage for the 142 wheat cultivars

	Mutation burden			Gene		Mutation burden			Gene
Sequence sample label	Heterozygous	Homozygous	Total	expression	Sequence sample label	Heterozygous	Homozygous	Total	expression
001_Preston	0.0447	0.0930	0.1377	9.3161	072_ACSplendor	0.0508	0.1075	0.1583	10.9015
002_Kota	0.0518	0.0860	0.1378	9.9271	073_Journey	0.0486	0.1006	0.1492	9.8010
003_Supreme	0.0458	0.0927	0.1385	9.8220	074_Thatcher	0.0493	0.1036	0.1529	10.1388
004_BroatchsWhitehead	0.0447	0.0913	0.1360	9.3442	075_Coronation	0.0498	0.1075	0.1573	10.1923
005_Ceres	0.0493	0.0880	0.1373	10.2093	076_Saunders	0.0470	0.1016	0.1486	9.9008
006_Reliance	0.0452	0.0887	0.1339	10.8637	077_Chinook	0.0569	0.1066	0.1635	9.6098
007_Renown	0.0534	0.1052	0.1587	9.5831	078_Neepawa	0.0473	0.1026	0.1499	10.0631
008_Lee	0.0516	0.1079	0.1595	9.8287	079_Roblin	0.0503	0.1046	0.1549	10.1603
009_Lake	0.0463	0.1056	0.1519	9.9243	080_CDCMakwa	0.0450	0.1029	0.1479	11.1909
010_Columbus	0.0498	0.1036	0.1534	9.4577	081_ACSuperb	0.0465	0.1023	0.1487	9.7888
011_Leader	0.0511	0.1138	0.1650	9.4522	082_Stettler	0.0475	0.0953	0.1428	10.2303
012_Lancer	0.0510	0.1079	0.1588	9.0150	083_Katepwa	0.0460	0.1052	0.1512	10.1680
013_Pasqua	0.0472	0.1059	0.1530	10.8013	084_Lovitt	0.0447	0.1009	0.1456	9.2951
014_Prodigy	0.0453	0.0946	0.1400	9.4823	085_Harvest	0.0477	0.1006	0.1482	9.4322
015_ACElsa	0.0472	0.1026	0.1497	9.3120	086_CDCGo	0.0488	0.0996	0.1484	9.8610
016_ACCadillac	0.0463	0.1009	0.1473	9.9055	087_CDCAlsask	0.0481	0.1059	0.1540	9.7547
017_McKenzie	0.0443	0.1042	0.1486	10.5735	088_Somerset	0.0521	0.1175	0.1696	9.8609
018_ACIntrepid	0.0524	0.1032	0.1557	10.0698	089_Helios	0.0477	0.1128	0.1605	9.1525
019_ACAbbey	0.0506	0.1069	0.1575	9.3196	090_Carberry	0.0467	0.1052	0.1519	9.9567
020_CDCBounty	0.0500	0.0999	0.1499	10.1375	091_Muchmore	0.0488	0.1112	0.1600	10.0051
021_BW776Lillian	0.0538	0.1135	0.1673	9.3956	092_CDCKernen	0.0468	0.1092	0.1560	10.1778
022_Alvena	0.0503	0.1069	0.1572	10.0570	093_CDCStanley	0.0506	0.1069	0.1575	10.1239
023_AACBailey	0.0503	0.1059	0.1562	9.6745	094_CDCThrive	0.0452	0.1049	0.1501	9.5126
024_CDCVRMorris	0.0467	0.0973	0.1439	10.1684	095_CDCUtmost	0.0453	0.1052	0.1506	9.3343
025_AACElie	0.0473	0.1009	0.1482	9.5035	096_Vesper	0.0450	0.1069	0.1519	9.2153
026_Peace	0.0490	0.1019	0.1509	9.1727	097_CDCAlsask	0.0480	0.1026	0.1506	10.2800
027_Fieldstar	0.0452	0.1046	0.1497	10.1723	098_ACBarrie	0.0483	0.1056	0.1539	11.8439
028_Infinity	0.0455	0.0973	0.1428	9.2581	099_GoodeveBW841	0.0520	0.1082	0.1602	11.7744
029_Waskada	0.0477	0.1009	0.1486	9.9416	100_AACBrandon	0.0458	0.1009	0.1468	9.4804
030_CDCAbound	0.0483	0.1036	0.1519	9.3914	101_AACPenhold	0.0452	0.1006	0.1458	9.2561
031_RedFife	0.0433	0.1095	0.1529	10.3720	102_AACConnery	0.0511	0.1195	0.1706	9.2299
032_Ladoga	0.0500	0.1079	0.1578	9.3189	103_AACRedberry	0.0437	0.1072	0.1509	8.4447
033_Stanley	0.0510	0.1066	0.1575	9.6402	104_AACViewfield	0.0460	0.1059	0.1519	9.0681
034_Huron	0.0495	0.1085	0.1580	10.0970	105_AACConcord	0.0521	0.1125	0.1646	9.6373
035_Percy	0.0458	0.1039	0.1497	9.7143	106_AACAlida	0.0478	0.1056	0.1534	9.3224
036_WhiteFife	0.0452	0.1079	0.1530	10.8434	107_AACTisdale	0.0485	0.1006	0.1491	8.4489
037_Marquis	0.0443	0.0923	0.1367	9.9324	108_AACGoodwin	0.0463	0.1052	0.1516	8.7542
038_Prelude	0.0465	0.0999	0.1464	10.4086	109_AACStarbuck	0.0463	0.1046	0.1509	10.0297
039_Ruby	0.0490	0.0933	0.1423	9.5509	110_AACWheatland	0.0467	0.1039	0.1506	9.6133
040_EarlyTriumph	0.0477	0.1009	0.1486	9.0698	111_AACCirrus	0.0465	0.1042	0.1507	9.8573
041_Renfrew	0.0438	0.0960	0.1398	10.0658	112_AACBroadacres	0.0472	0.1059	0.1530	8.6108
042_Garnet	0.0496	0.0976	0.1473	9.2984	113_AACRussell	0.0491	0.1095	0.1587	9.1607
043_RedBobs222	0.0486	0.0963	0.1449	9.3868	114_AACHockley	0.0490	0.1049	0.1539	9.0664
044_Reward	0.0457	0.0960	0.1416	9.1393	115_AACRimbey	0.0462	0.0973	0.1434	10.9417
045_Canus	0.0455	0.1003	0.1458	10.0865	116_AACCrossfield	0.0478	0.1105	0.1583	9.9347
046_Apex	0.0447	0.0996	0.1443	9.3184	117_AACEntice	0.0513	0.1085	0.1598	10.2584
047_Regent	0.0467	0.1049	0.1516	8.7834	118_AACWestlock	0.0470	0.1029	0.1499	9.5464
048_Rescue	0.0521	0.1059	0.1580	9.4898	119_AACPerform	0.0477	0.1099	0.1575	10.1003
049_Redman	0.0950	0.0576	0.1525	9.4262	120_Unity	0.0460	0.1039	0.1499	9.4522
050_Selkirk	0.0453	0.0993	0.1446	9.9661	121_Shaw	0.0521	0.0946	0.1468	8.7787
051_Canthatch	0.0430	0.1046	0.1476	10.3440	122_Cardale	0.0490	0.0983	0.1473	9.7724
052_Pembina	0.0470	0.0973	0.1443	10.2378	123_AACRedwater	0.0510	0.1089	0.1598	9.4365
053_Cypress	0.0490	0.0960	0.1449	9.7085	124_AACPrevail	0.0473	0.1049	0.1522	8.8848
054_Park	0.0447	0.0983	0.1430	9.7678	125_AACCameron	0.0440	0.1032	0.1473	9.1249
055_Manitou	0.0465	0.1036	0.1501	10.2377	126_AACJatharia	0.0448	0.1085	0.1534	8.6906
056_Canuck	0.0490	0.0976	0.1466	9.9266	127_AACTradition	0.0472	0.1026	0.1497	9.5768
057_Sinton	0.0470	0.1095	0.1565	9.8855	128_AACWarman	0.0450	0.1049	0.1499	10.0958
058_Chester	0.0481	0.1125	0.1607	9.9516	129_AACLeRoy	0.0452	0.1052	0.1504	10.2506
059_Benito	0.0486	0.1032	0.1519	10.6145	130_AACMagnet	0.0465	0.1138	0.1603	8.5524
060_Kenyon	0.0453	0.1019	0.1473	9.3335	131_AACHodge	0.0508	0.1115	0.1623	8.1111
061_Conway	0.0430	0.1052	0.1482	9.8792	132_AACRedstar	0.0490	0.1085	0.1575	9.8003
062_Laura	0.0496	0.1016	0.1512	9.4619	133_CDCImagine	0.0462	0.1072	0.1534	9.0352
063_CDCTeal	0.0496	0.1049	0.1545	10.1233	134_CDCPlentiful	0.0467	0.1013	0.1479	9.5956
064_CDCMerlin	0.0495	0.1039	0.1534	10.5301	135_CDCBradwell	0.0467	0.1039	0.1506	8.9977
065_ACMichael	0.0477	0.1029	0.1506	10.5142	136_CDCHughes	0.0493	0.1042	0.1535	9.4394
066_ACEatonia	0.0534	0.1122	0.1656	9.6600	137_CDCCordonCLPlus	0.0516	0.1118	0.1635	8.6751
067_ACDomain	0.0453	0.1023	0.1476	10.4366	139_CDCLandmark	0.0462	0.1036	0.1497	9.7929
068_Invader	0.0486	0.1042	0.1529	9.5926	140_CDCOrtona	0.0498	0.1079	0.1577	9.4225
069_ACCora	0.0440	0.1085	0.1525	11.1748	141_CDCSkrush	0.0472	0.0993	0.1464	9.6800
070_Pacific	0.0478	0.1032	0.1511	10.1732	142_CDCPilarCLPlus	0.0473	0.1112	0.1585	9.0045
071_ACMajestic	0.0491	0.1046	0.1537	10.2554	143_CDCSuccessionCLPlus	0.0477	0.1072	0.1549	9.9214

Fig. S1. The distributions of all identified SNPs (A and C) and deleterious SNPs (B and D) across 21 oat and 21 wheat chromosomes for 141 oat and 142 wheat cultivars, respectively. The identified deleterious SNPs in oat and wheat cultivars were widely distributed across their 21 chromosomes

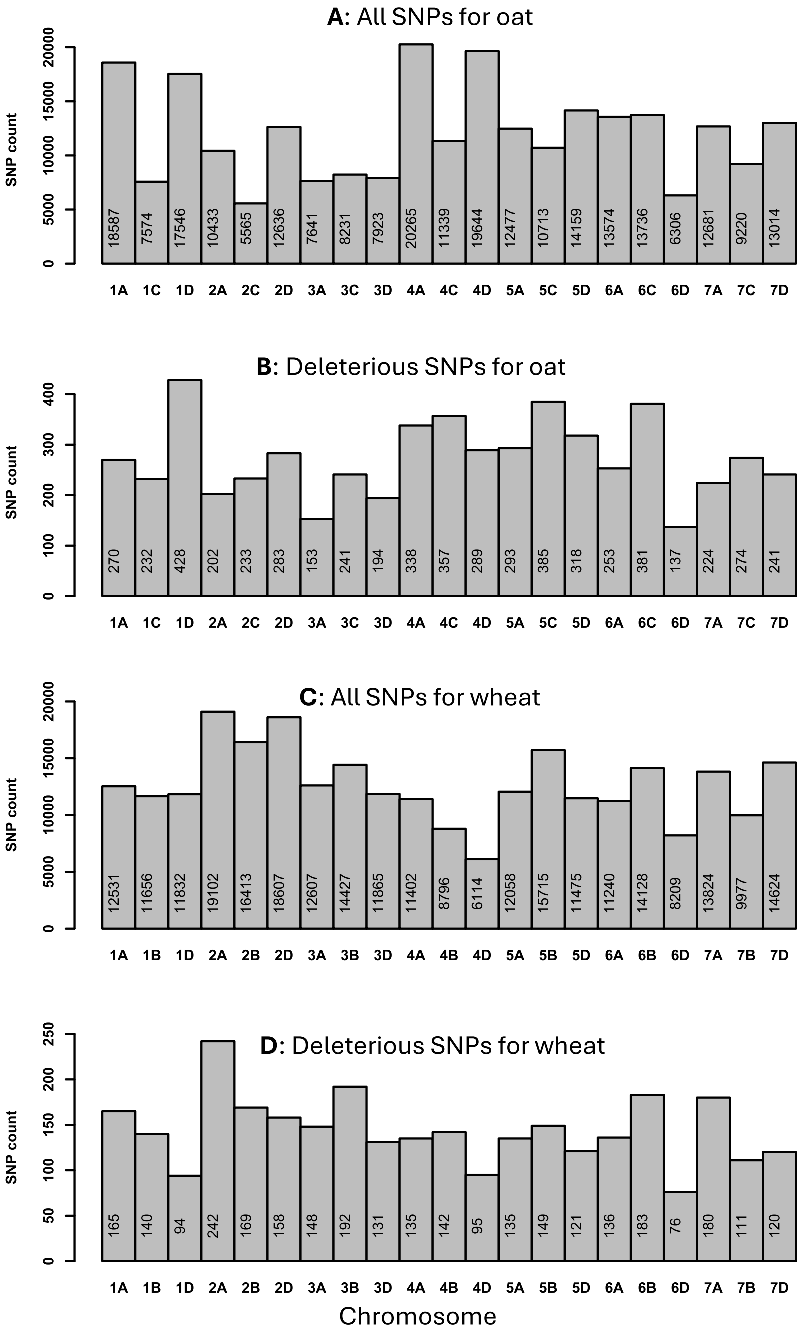

Fig. S2. The distributions of minor allele frequencies for all SNP data (A) and deleterious SNP data (B) in 141 oat and 142 wheat cultivars. The distribution patterns are expected, except the considerable heterozygous SNP genotypes observed in the wheat cultivars (as shown in A2)

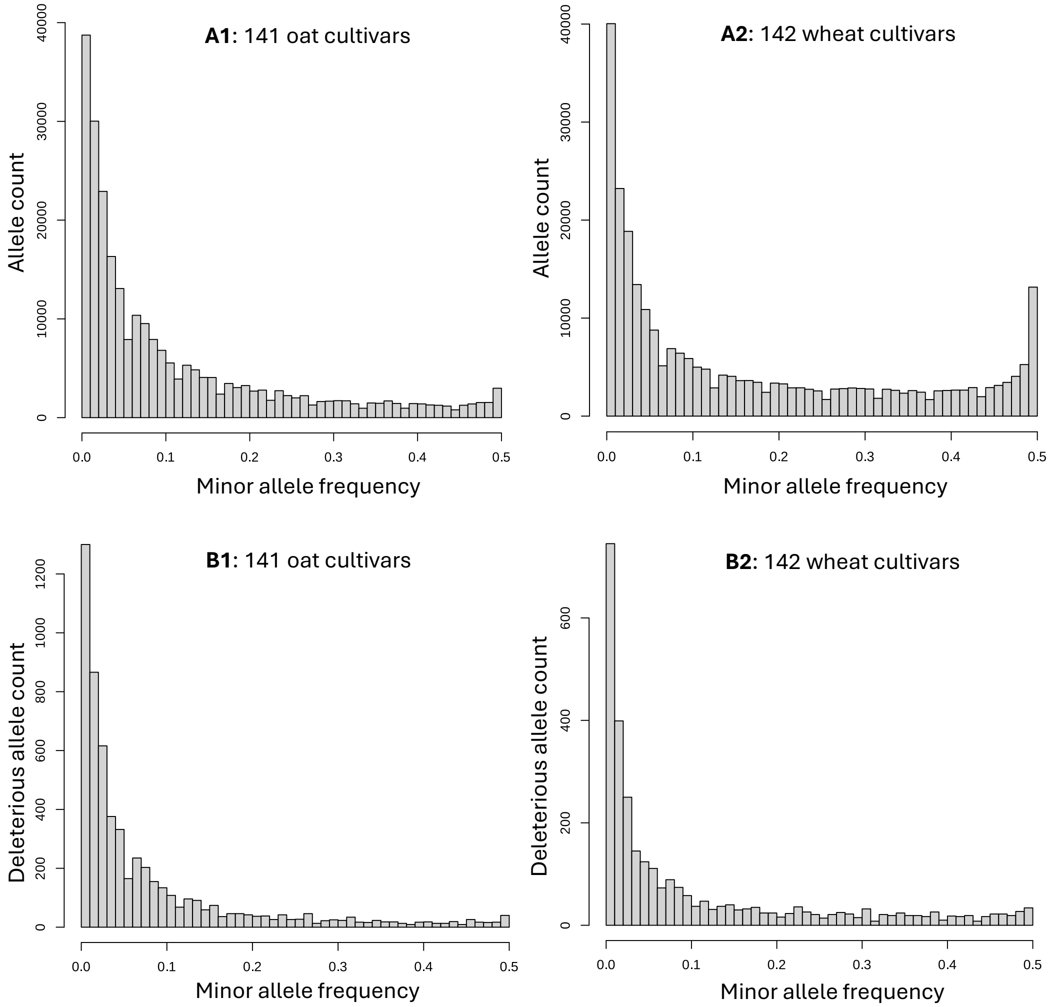

Fig. S3. The distributions of GERP++ RS scores for the identified deleterious SNPs in oat and wheat cultivars, showing the severity of their possible detrimental impacts. SNPs with RS scores <1, 1-3, or >3 would be classified as weakly, mildly, or highly deleterious, respectively. There were 1083 and 27 highly deleterious oat and wheat SNPs, respectively

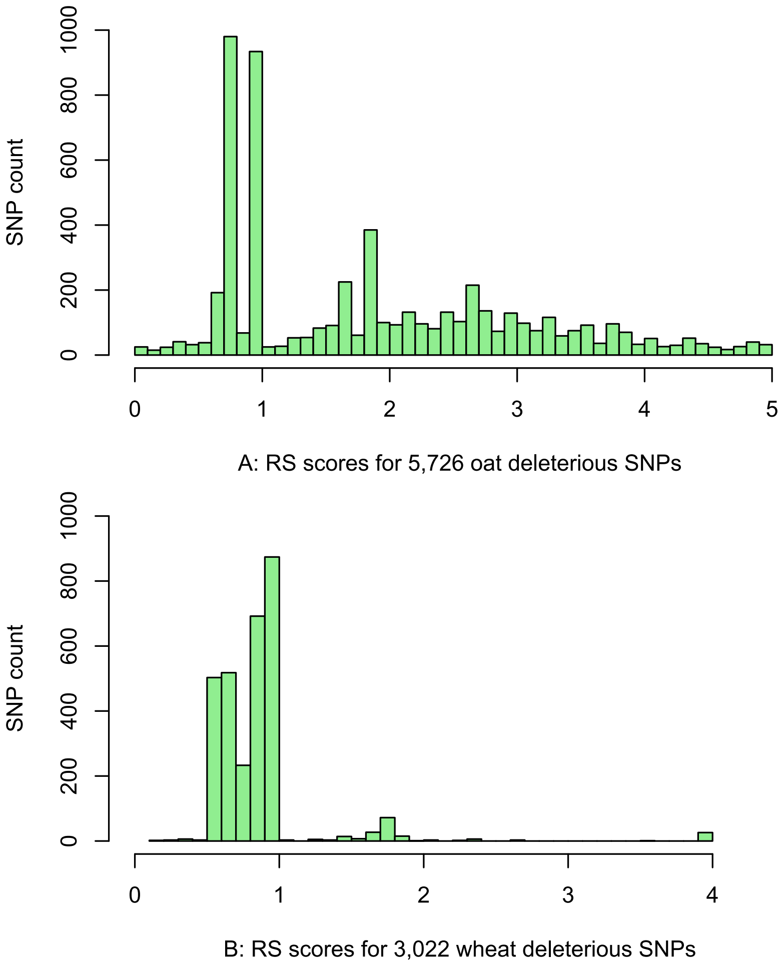

Fig. S4. REVIGO gene ontology treemap showing cluster representatives of the 537 biological processes associated with significant 585 GO terms that were extracted from 7,157 genes associated with the oat deleterious SNPs

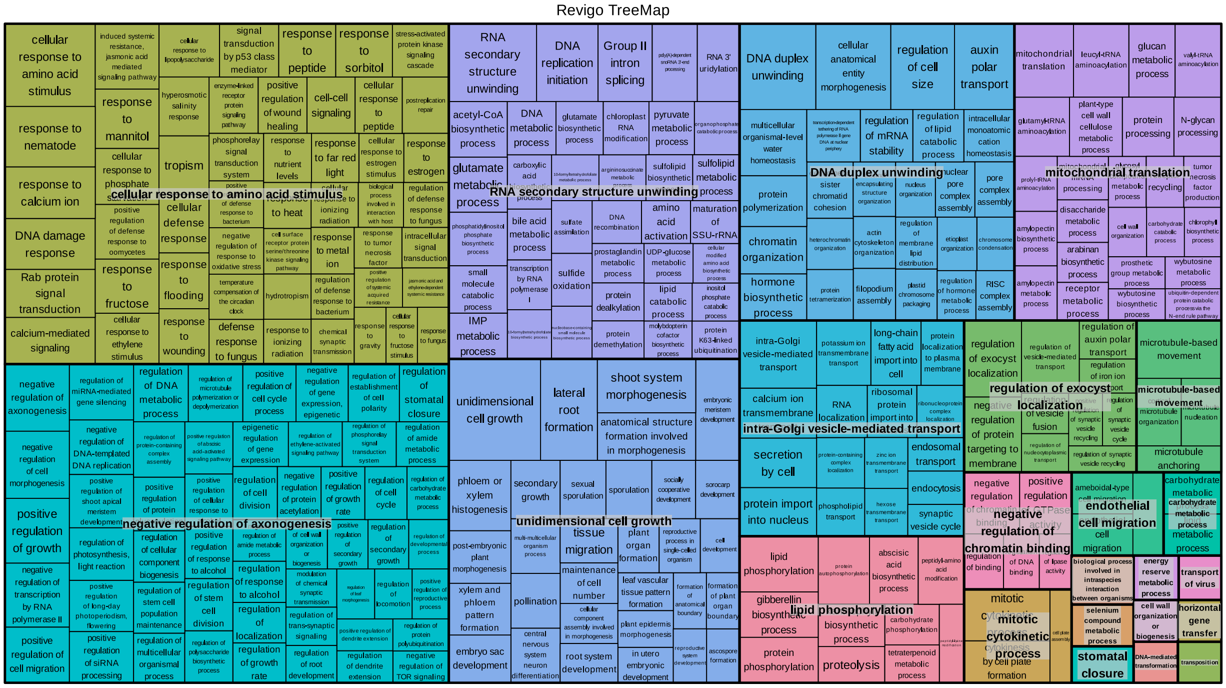

Fig. S5. REVIGO gene ontology treemap showing cluster representatives of the 232 biological processes associated with significant 262 GO terms that were extracted from 3,533 genes associated with the wheat deleterious SNPs

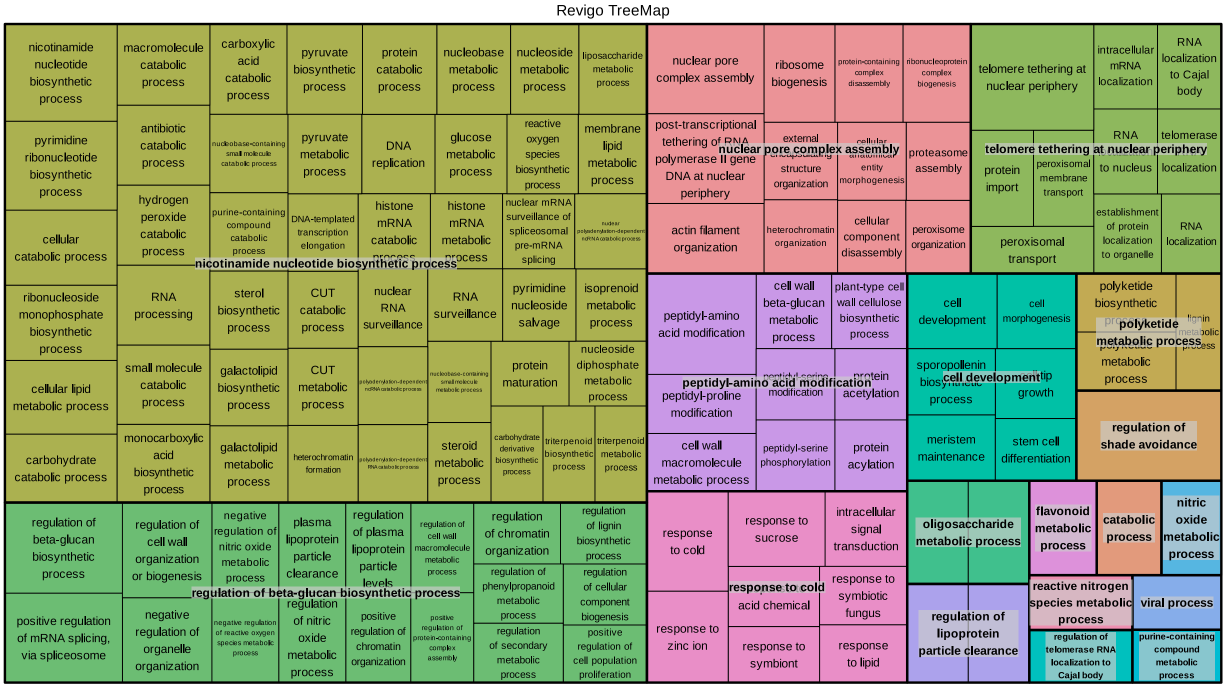

Fig. S6. Top 40 cellular components inferred with g:Profiler from significant GO terms of 7,157 oat (A) and 3,533 wheat (B) genes associated with the identified deleterious SNPs. The 19 cellular components shared between oat and wheat were highlighted in brown

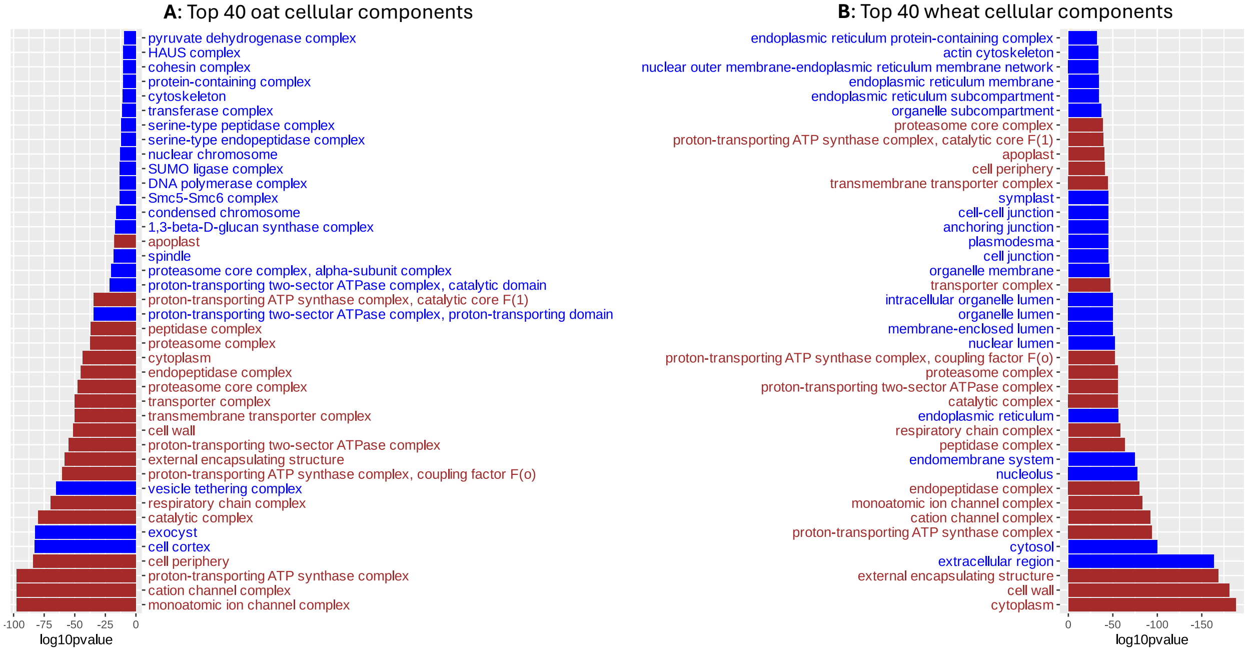

Fig. S7. Top 40 molecular functions inferred with g:Profiler from significant GO terms of 7,157 oat (A) and 3,533 wheat (B) genes associated with the identified deleterious SNPs. The four molecular functions shared between oat and wheat were highlighted in brown

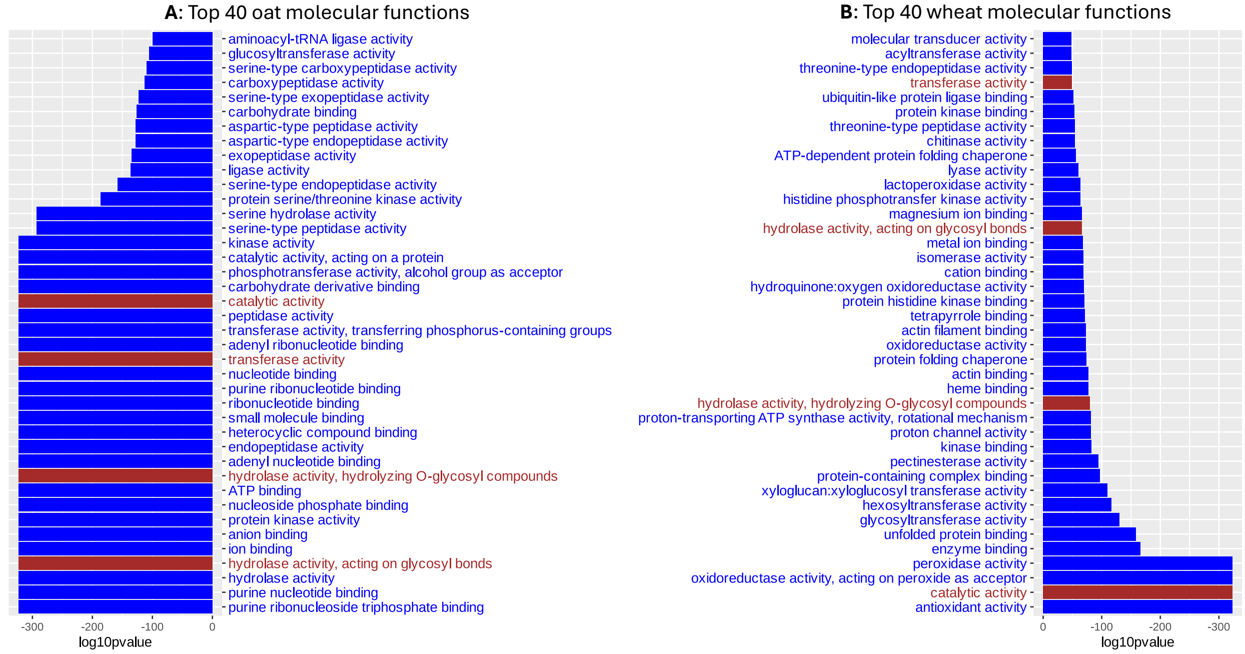

Fig. S8. The patterns of gene expressions associated with the identified deleterious SNPs in early seedling growth over the registration years of 141 oat (A) and 142 wheat (B) cultivars, as measured with average transcripts per million per gene. When the wheat cultivar released in 1845 was excluded, the linear regression of gene expressions over the 141 wheat cultivar registration years remained not statistically significant (the coefficient=-0.0024, R^2=0.0124, P=0.10)

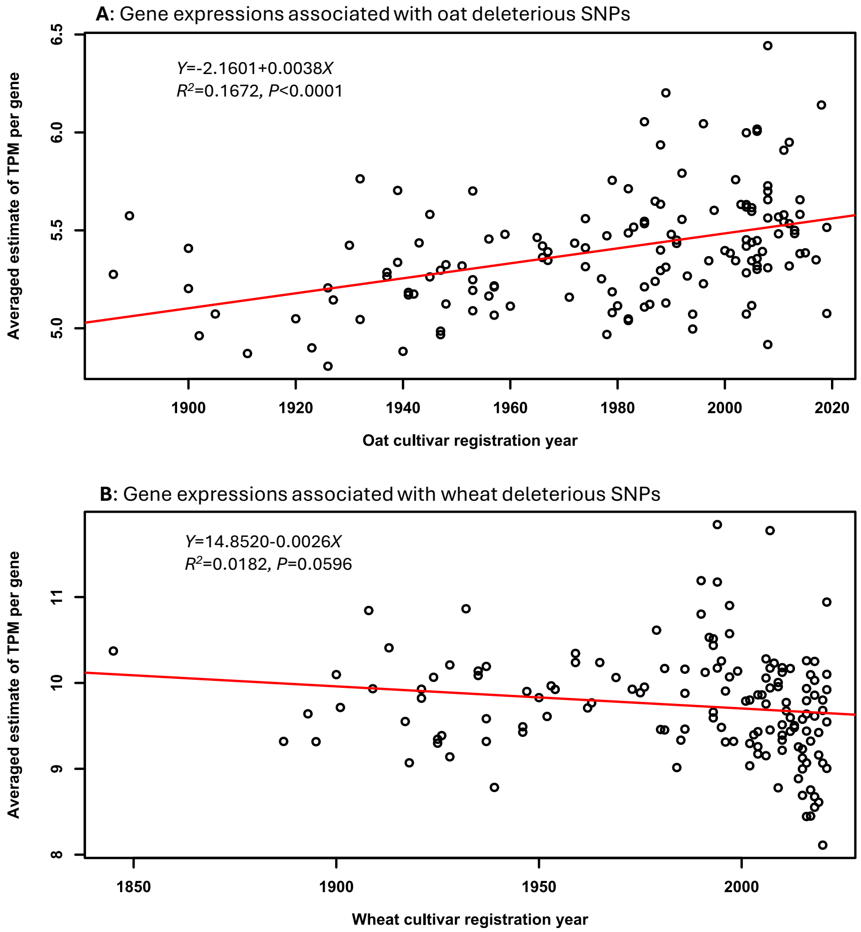

Fig. S9. The ranking patterns of estimated three mutation burdens per deleterious locus (total, heterozygous, homozygous) for 141 oat (A) and 142 wheat (B) cultivars

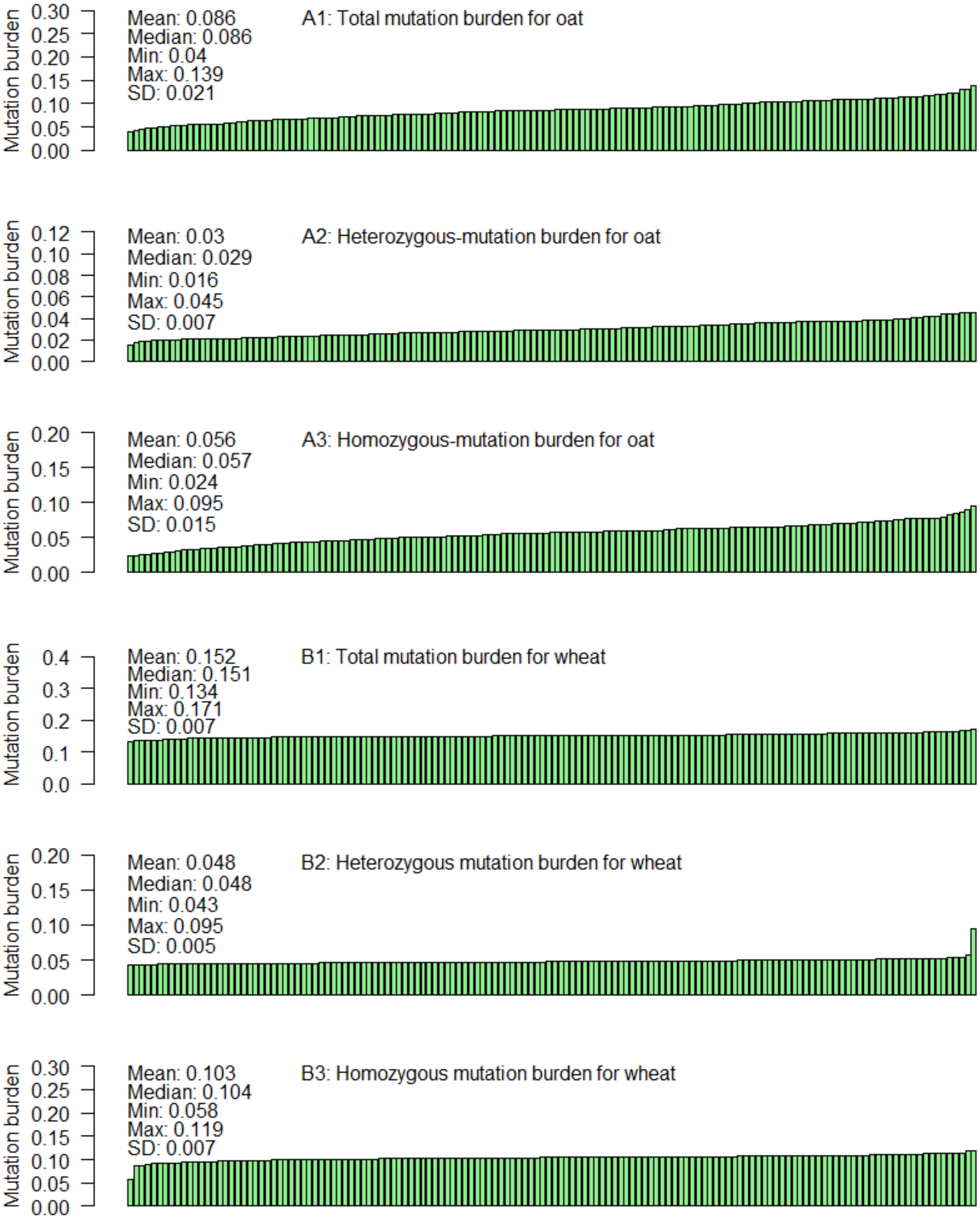

Supplement: Supplementary file 1 [file cells-14-00844-s001.zip › cells-3672128-supplementary.pdf]
